# Small Fluorogenic Amino Acids for Peptide‐Guided Background‐Free Imaging

**DOI:** 10.1002/anie.202216231

**Published:** 2022-12-14

**Authors:** Fabio de Moliner, Zuzanna Konieczna, Lorena Mendive‐Tapia, Rebecca S. Saleeb, Katie Morris, Juan Antonio Gonzalez‐Vera, Takeshi Kaizuka, Seth G. N. Grant, Mathew H. Horrocks, Marc Vendrell

**Affiliations:** ^1^ Centre for Inflammation Research The University of Edinburgh UK; ^2^ EaStCHEM School of Chemistry The University of Edinburgh UK; ^3^ Nanoscopy-UGR Laboratory Facultad de Farmacia Universidad de Granada Spain; ^4^ Centre for Clinical Brain Sciences The University of Edinburgh UK

**Keywords:** Fluorescence, Microscopy, Probes, Proteins, Super-Resolution

## Abstract

The multiple applications of super‐resolution microscopy have prompted the need for minimally invasive labeling strategies for peptide‐guided fluorescence imaging. Many fluorescent reporters display limitations (e.g., large and charged scaffolds, non‐specific binding) as building blocks for the construction of fluorogenic peptides. Herein we have built a library of benzodiazole amino acids and systematically examined them as reporters for background‐free fluorescence microscopy. We have identified amine‐derivatized benzoselenadiazoles as scalable and photostable amino acids for the straightforward solid‐phase synthesis of fluorescent peptides. Benzodiazole amino acids retain the binding capabilities of bioactive peptides and display excellent signal‐to‐background ratios. Furthermore, we have demonstrated their application in peptide‐PAINT imaging of postsynaptic density protein‐95 nanoclusters in the synaptosomes from mouse brain tissues.

## Graphical abstract

Benzodiazole amino acids are excellent small building blocks for the construction of background‐free peptide probes for fluorescence imaging. We demonstrate their robustness and versatility for solid‐phase peptide synthesis, their minimally invasive character, and their compatibility with different optical imaging modalities, including super‐resolution peptide‐PAINT imaging.

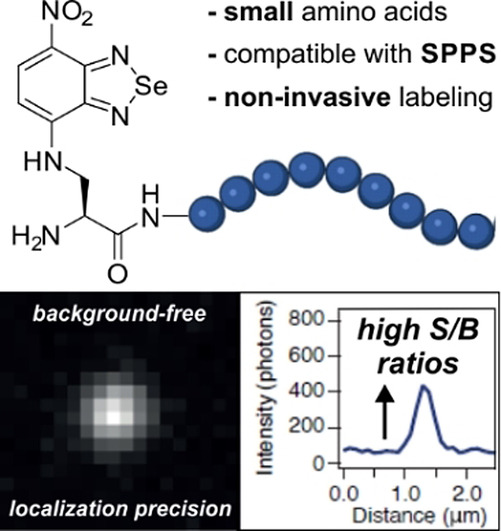

## Introduction

Fluorescence microscopy is one of the most common and versatile techniques to examine the localization and trafficking of proteins with high spatiotemporal resolution.^[1]^ However, the wave nature of light and its associated diffraction limit the resolution of optical microscopy to ≈250 nm. New methods, collectively grouped under the term super‐resolution (SR) microscopy, can overcome this physical limit, gaining over an order of magnitude in precision.^[2]^ Alongside the development of methods for acquisition and image analysis, an increasing number of fluorophores for SR microscopy have been reported.^[3]^ Different structures including cyanines,^[4]^ ATTO dyes,^[5]^ and rhodamines^[6]^ among others exhibit suitable optical properties. However, these fluorophores often feature charged scaffolds with molecular weights over 500 Da, and their incorporation into biomolecules (e.g., peptides) typically requires chemical spacers to minimize any potential interference in bioactivity and molecular recognition. As a result, there is a high demand for small fluorophores that can accelerate the development of peptide‐based probes for targeted fluorescence microscopy.

Several SR methods rely on the stochastic separation of the emission of single fluorophores in time to gain spatial resolution, including photo‐activation localization microscopy (PALM), stochastic optical reconstruction microscopy (STORM) and point accumulation for imaging in nanoscale topography (PAINT).^[7]^ In recent years, PAINT imaging has gained increased attention due to its versatility. In its original implementation, PAINT uses fluorophores that are emissive upon target binding, with initial reports describing lipophilic dyes that emit strongly in hydrophobic regions.^[8]^ As an alternative to environmentally‐sensitive dyes, photoactivatable fluorophores have been used in interface‐PAINT^[9a]^ and reservoir‐PAINT.^[9b]^ More recently, PAINT has been adapted to DNA and peptide structures for imaging non‐lipidic structures. DNA‐PAINT can be used to image biomolecules after transient hybridization with complementary fluorophore‐tagged oligonucleotides;^[10]^ however, it is constrained by the slow binding rate of complementary oligonucleotides, which are typically in the order of 10^6^ M^−1^ s^−1^. In practical terms, this means that high resolution DNA‐PAINT images can take several hours to acquire, although the use of quenchers in fluorogenic DNA‐PAINT,^[11a]^ or Förster Resonance Energy Transfer (FRET)‐based probes in FRET‐PAINT^[11b]^ has reduced this time by an order of magnitude. In the last years, Jungmann et al. have reported peptide‐PAINT as an alternative modality with improved speed and efficiency.^[12]^ Peptide‐PAINT exploits the rapid, specific, and transient interactions between peptides and proteins to produce SR images with faster kinetics than DNA‐PAINT.^[13]^ Recently, Oi and co‐workers have described Live cell Imaging using reVersible intEractions (LIVE)‐PAINT for SR microscopy in live cells;^[13c]^ however, most peptide‐PAINT and LIVE‐PAINT reporters employ fluorophores with similar shortcomings to other SR dyes (e.g., hydrophobicity, non‐specific binding, charged structures). We envisioned that smaller and neutral amino acids would accelerate the design of peptide‐based probes for background‐free imaging, including PAINT microscopy.

Our group and others have described unnatural fluorescent amino acids and integrated them into peptides and proteins to generate live‐cell imaging reporters.^[14]^ Despite the emerging use of fluorescent amino acids in optical probes, there are few systematic reports of their optimization as generic building blocks for peptide‐guided imaging. In this work, we have built a collection of nitrobenzodiazole amino acids and examined their suitability as reporters for wash‐free fluorescence microscopy. From our library, we identified chemically stable amino acids that can be scaled up and used in solid‐phase peptide synthesis (SPPS) to prepare optimal probes for PAINT imaging. Benzodiazole amino acids retain the molecular recognition properties of native peptides and provide fluorescence microscopy images with high signal‐to‐background (S/B) ratios. Notably, we demonstrated the versatility of this approach by producing different fluorescent peptide sequences and demonstrating their application for targeted imaging of subcellular structures in the synaptosomes of mouse brain tissues. To the best of our knowledge, these are the first small amino acids for background‐free peptide‐PAINT imaging.

## Results and Discussion

### Chemical Synthesis and Photophysical Characterization of Nitrobenzodiazole Amino Acids

**Figure 1:**
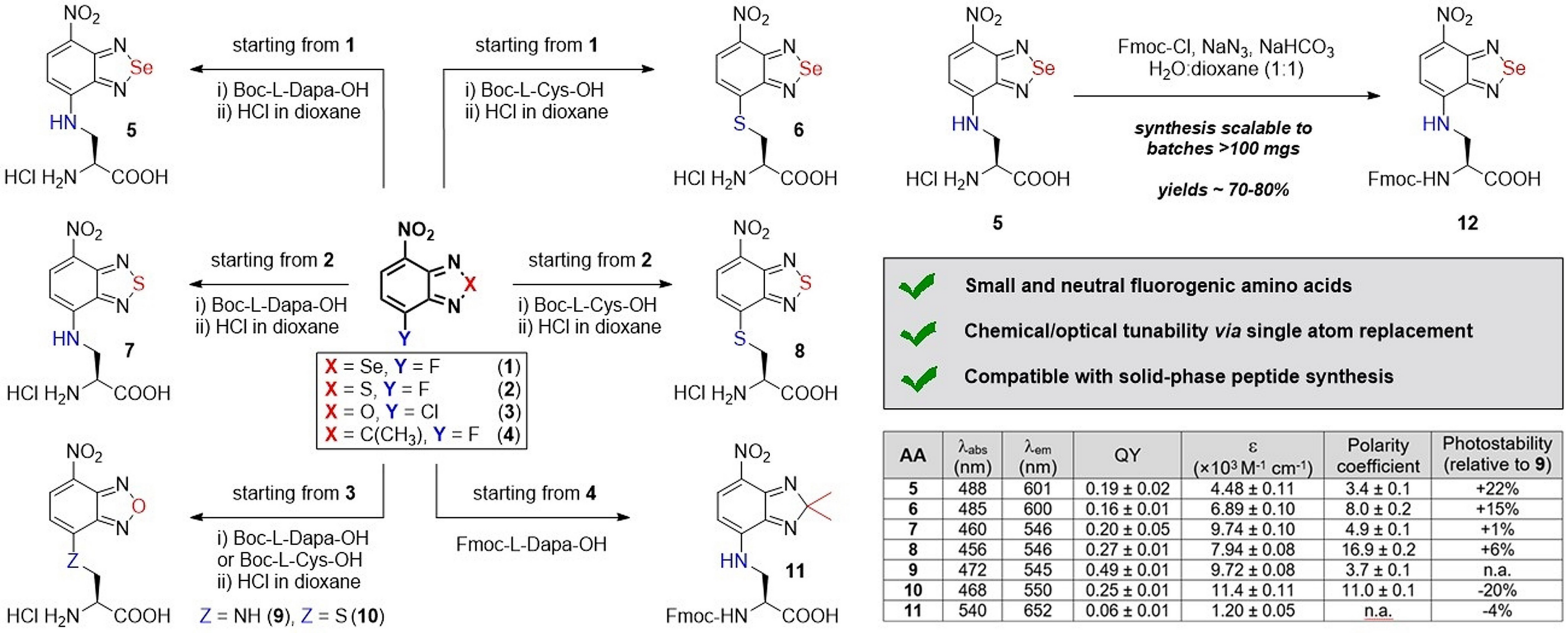
Chemical synthesis of nitrobenzodiazole amino acids. The table summarizes the optical properties of all amino acids. Relative quantum yields (QY) were determined in DMSO using rhodamine B as a standard.

Building from the work of our group and others in the derivatization of benzodiazoles as tunable fluorophores,^[15]^ we designed a new collection of amino acids where we systematically introduced modifications in the positions 4 and 7 of the nitrobenzodiazole core. Because these positions directly contribute to the push‐pull dipole of the fluorophore,^[16]^ we envisaged that the introduction of different atoms would lead to variations in the optical behavior of the amino acids. We prepared Se‐ and S‐containing heterocycles (**1** and **2**, Figure 1) from the commercially available 2‐fluoro‐*o*‐phenylenediamine via a two‐step approach comprising: 1) incorporation of the bridging heteroatom from SeO_2_ (for compound **1**) or N‐sulfinylaniline (for compound **2**), and 2) nitration in acidic conditions. The resulting fluoronitrobenzodiazoles were conjugated to Boc‐L‐diaminopropionic acid (Dapa) or Boc‐L‐cysteine (Cys) to introduce amine or thiol‐based electron donating groups that favored a push‐pull dipole. First, compounds **1** and **2** were subjected to nucleophilic aromatic substitution with Dapa or Cys under basic conditions and mild heating, and later we obtained the free amino acids (**5**–**8**) by removal of the Boc groups under acid conditions. This synthetic approach displayed overall yields >80 % and required one purification step prior to Boc removal.

Furthermore, this strategy proved to be compatible with batches ≈100 mg and suitable for the preparation of building blocks for SPPS. For the synthesis of compound **11**, a different route was needed, mostly due to the acid lability of C‐bridged nitrobenzodiazoles.^[17]^ In this case, 2‐fluoro‐5‐nitro‐*o*‐phenylene‐diamine was obtained by removal of the Se atom from compound **1** and utilized in a Cu‐catalyzed condensation with acetone to render the C‐bridged benzodiazole **4**. The final nucleophilic aromatic substitution with Fmoc‐L‐Dapa‐OH rendered the fluorescent amino acid **11** in moderate yields (24 %). Importantly, as a control for the optical characterization assays, we also synthesized Dapa and Cys derivatives of the O‐bridged nitrobenzoxadiazole (NBD, compounds **9**–**10** in Figure 1) by coupling the commercial precursor **3** to the Boc‐protected amino acids and subsequent deprotection with yields around 80 %. Finally, we also scaled up the synthesis of Fmoc‐protected analogues by treatment of the free amino acids with Fmoc‐azide ‐obtained by reaction between Fmoc‐Cl and sodium azide‐ with good yields (i.e., 75 % for compound **12**, Figure 1).

Chemically stable fluorescent building blocks are crucial for the automated synthesis of peptide‐based probes because they can minimize conjugation and purification steps and accelerate the preparation of high‐throughput and multiplexed libraries. Therefore, we examined the chemical stability of amino acids **5**–**11** by HPLC‐MS under some of the most common reaction conditions used in SPPS (Table 1 and Supporting Information for details). Unlike other environmentally‐sensitive fluorescent amino acids,^[18]^ most nitrobenzodiazole amino acids were stable in 95 % trifluoroacetic acid (TFA) for over 1 h, with the exception of the acid‐labile compound **11**. Furthermore, Dapa derivatives (compounds **5**, **7** and **9**) showed good compatibility with most reagents (e.g., piperidine, tetrabutylammonium fluoride (TBAF), hydrazine, Pd^0^), whereas Cys analogues (compounds **6**, **8** and **10**) showed limited stability in TBAF and hydrazine. Of note, we did not attempt hydrogenation reactions because the nitro groups in benzodiazole amino acids can be readily reduced to the corresponding amines, resulting in weaker push‐pull dipoles and shorter emission wavelengths. Altogether, these results demonstrate the compatibility of amine‐derivatized nitrobenzodiazole amino acids with most protocols used in SPPS.

**Table 1:** Chemical stability of amino acids **5**–**11** to standard SPPS reagents.

AA	TFA 95 %^[a]^	Piperidine DMF^[b]^	TBAF^[c]^	Pd^0[d]^	N_2_H_4_^[e]^
5	>95 %	>95 %	>95 %	>95 %	>95 %
6	>95 %	51±8 %	<5 %	47±8 %	<5 %
7	>95 %	84±4 %	>95 %	92±1 %	65±7 %
8	>95 %	10±2 %	<5 %	<5 %	<5 %
9	>95 %	89±2 %	79±3 %	>95 %	<5 %
10	>95 %	25±8 %	<5 %	<5 %	<5 %
11	<5 %	n.a.	>95 %	65±5 %	75±2 %

Reaction conditions: [a] TFA:DCM (95 : 5), 1 h, r.t.; [b] Piperidine:DMF (2 : 8), 30 min, r.t.; [c] TBAF 3 H_2_O (3 eq.) in DCM, 1 h, r.t., [d] Pd(PPh_3_)_4_ (0.25 eq.) in DCM, 30 min, r.t., [e] 10 % N_2_H_4_ H_2_O in DMF, 1 h, r.t. HPLC purities (254 nm) are presented as means±SD (n=3).

Next, we measured the optical properties of benzodiazole amino acids to assess their suitability as background‐free reporters for fluorescence microscopy (Figure 1) and compared them to conventional PAINT dyes (Figure S1). Absorbance and emission maxima wavelengths spanned from 456 to 540 nm and from 545 to 652 nm respectively, with notable solvatochromic behavior (Figure S2) in line with reports of similar structures.^[19]^ Se‐bridged amino acids **5** and **6** showed around 60 nm longer emission maxima than the S‐bridged compounds **7** and **8** and the NBD amino acids **9** and **10**. Fluorescence quantum yields and extinction coefficients were in the same order of magnitude for all amino acids (between 16 % and 49 %), except for compound **11**, which displayed the weakest brightness among all derivatives. Even though the C‐bridging group led to the longest emitting amino acid (>650 nm), the poor brightness of this fluorophore makes it impractical for peptide‐guided imaging. We also measured the fluorescence sensitivity of the amino acids to environments of variable polarity and viscosity and compared their optical readouts to the standard NBD. First, we assessed the polarity dependence by measuring their emission in water‐dioxane mixtures. Compounds **5**–**8** showed very low background signals in aqueous media and strong fluorogenic responses in non‐polar environments, showing similar or higher polarity coefficients than NBD and good potential for wash‐free imaging. We also performed viscosity experiments in mixtures of glycerol and water, where most amino acids exhibited similar properties and minor turn‐on effects (Figure S3). Fluorescence lifetime measurements of the amino acids **5**–**8** also confirmed their remarkable environmental sensitivity, with negligible τ values in water (<1 ns) and higher values in non‐polar solvents, such as dioxane (e.g., from 5.2 ns to 9.6 ns, Figure S4).

Finally, we analyzed the photostability of all amino acids because NBD derivatives can be readily photobleached.^[20]^ Notably, the presence of Se in Dapa‐ and Cys‐functionalized benzodiazoles led to an increase in photostability, with compounds **5** and **6** being ≈20 % more photostable than the NBD amino acid **9**. On the other hand, minor differences in photostability were found between O‐bridged and S‐bridged benzodiazoles, with compound **8** being 6 % more photostable than compound **9**. Collectively, these results indicate the potential of nitrobenzodiazole amino acids as small, fluorogenic and photostable building blocks for the SPPS of multicolored peptide‐based probes.

### The Benzoselenadiazole Amino Acid 5 Displays Suitable Optical Properties for Background‐Free Fluorescence Imaging

**Figure 2:**
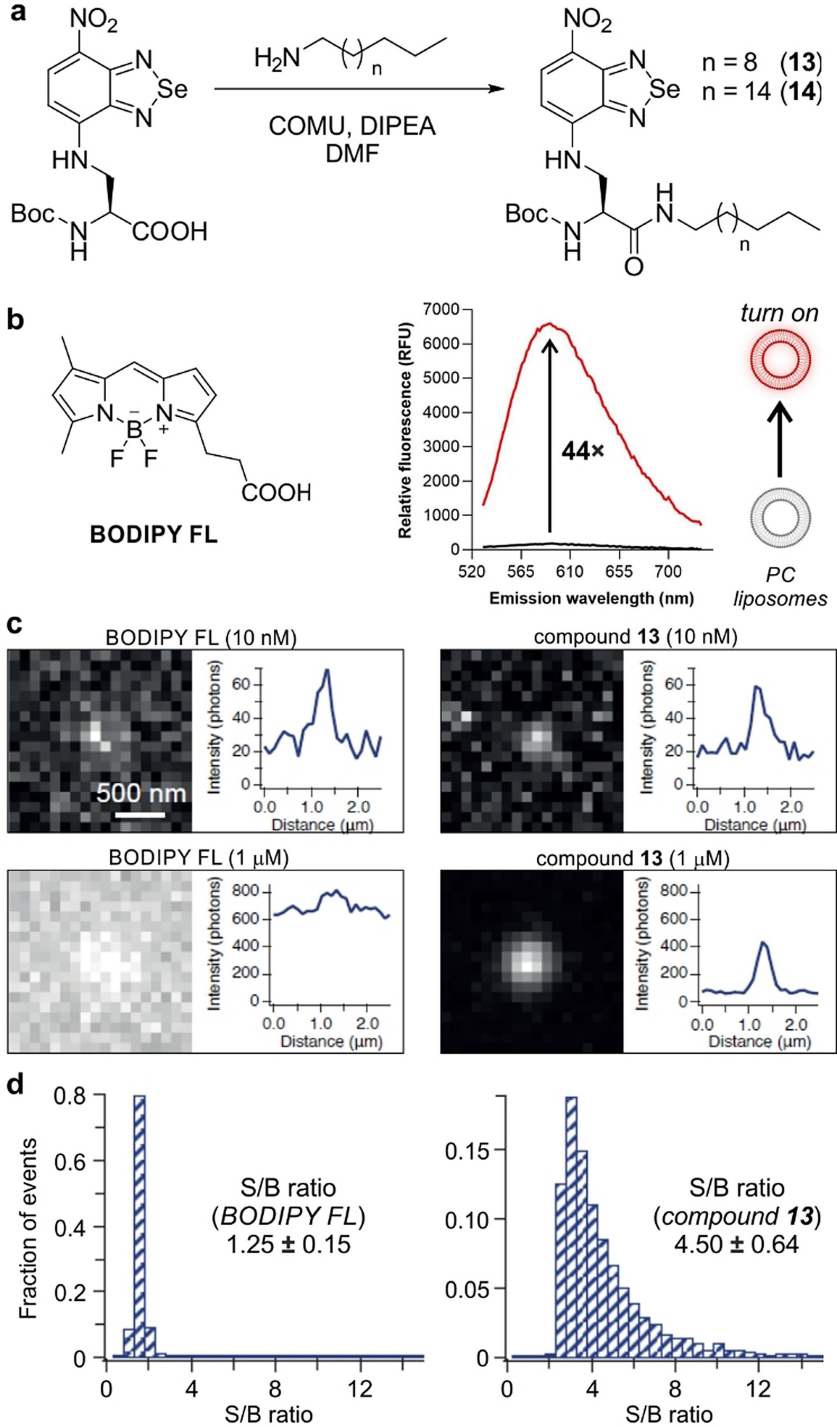
a) Chemical synthesis of lipid analogues of the benzoselenadiazole amino acid **5**. b) Representative spectra (from 3 experiments) of compound **13** (10 μM) in phosphate buffer saline (PBS, black) or in PBS containing phosphatidylcholine (PC)‐based liposomes (red). Excitation wavelength: 500 nm. c) Wash‐free microscopy images of liposomes after incubation with compound **13** or BODIPY‐FL at the indicated concentrations. d) Representative histograms of signal‐to‐background (S/B) ratios in PC liposomes after imaging with BODIPY‐FL or compound **13** (both 1 μM). Data as means±SD (n=8).

Given the properties of compound **5** as a chemically and photostable amino acid for peptide synthesis, we decided to examine its properties for fluorescence microscopy. Our initial characterization confirmed that the benzoselenadiazole core shows notable environmental sensitivity (see table in Figure 1), therefore we designed experiments to quantitatively measure its fluorescence emission in hydrophobic environments. For this purpose, we synthesized two lipid analogues that would favor the insertion of the fluorescent amino acid **5** in liposomes (Figure 2). Specifically, we employed the Boc‐protected analogue of the amino acid **5** to form carboxamides by coupling the carboxylic acid group to two alkylamines of different length (compounds **13** and **14**, Figure 2).

Both compounds were isolated in good yields and purities (full characterization in the Supporting Information), and showed very similar absorbance and emission maxima wavelengths to the original amino acid **5** (Figure S5). Compound **13** displayed higher fluorogenicity in phosphatidylcholine (PC)‐based liposomes (Figures 2 and S6), thus we employed it for further experiments using wash‐free microscopy. Although the exact reasons behind the enhanced fluorogenicity of compound **13** remain elusive, it is possible that the C_12_ alkyl chain inserted better into PC liposomes as similar trends have been reported in other fluorescent lipids.^[21]^ We incubated PC liposomes with different concentrations of compound **13** or BODIPY‐FL as a commercial lipophilic control dye, and imaged them on a total internal reflection fluorescence (TIRF) microscope under wash‐free conditions. The two compounds exhibited photobleaching lifetimes of the same order (e.g., 0.45±0.27 s for compound **13** and 1.25±0.88 s for BODIPY‐FL), with compound **13** exhibiting a notable turn‐on effect (e.g., intensity above background 291±48 photons for compound **13**, 192±16 photons for BODIPY‐FL) and higher S/B ratios (e.g., 4.50 for compound **13**, 1.25 for BODIPY‐FL). On the other hand, BODIPY‐labeled liposomes were only distinguishable at low concentrations and required washing steps at micromolar concentrations. These results highlight the minimal background of benzoselenadiazole amino acids and their suitability for wash‐free fluorescence imaging.

### Solid‐Phase Synthesis of 5‐Labeled Fluorescent Peptides and their Biophysical Characterization

After the identification of amino acid **5** as a suitable building block for wash‐free fluorescence microscopy, we assessed its application for peptide‐PAINT imaging. For this study, we focused on the complementary sequences 101A and 101B that were recently reported by DeLoache, Dueber and co‐workers as an α‐helix peptide pair with specific and transient interaction and binding affinities in the nM range.^[22]^ Given the small size and fluorogenicity of amino acid **5**, we envisaged that its incorporation into the 101A sequence would not impair the binding between the two peptides and enable direct in situ monitoring of their molecular interactions.

**Figure 3:**
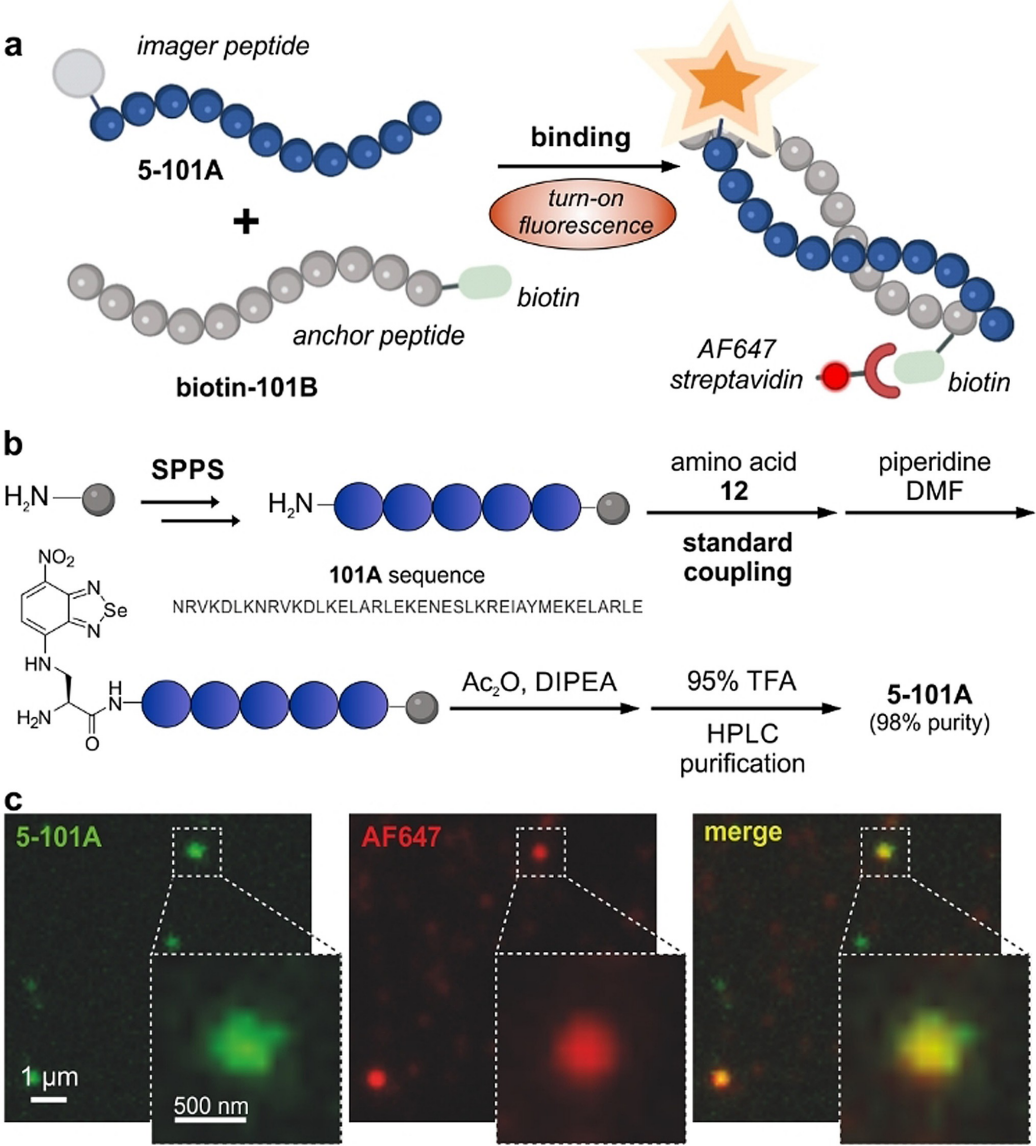
a) Schematic illustration of a complementary peptide imaging pair using the amino acid **5** as the direct binding reporter. The peptide **5‐101A** is regarded as the “imager peptide” and the peptide **biotin‐101B** as the “anchor peptide”. b) Synthetic scheme for the preparation of the peptide **5‐101A** using standard protocols in SPPS. For synthetic details, see Figure S7 and Supporting Information. c) Representative fluorescence microscopy images showing the binding between **5‐101A** (100 nM, green) and **biotin‐101B** (100 nM) in the presence of AlexaFluor647 (AF647)‐streptavidin (17 nM, red). Merge fluorescence signals displayed in yellow. Similar contrast was observed between fluorescence images with **5‐101A** (wash‐free) and fluorescence images with AF647‐streptavidin (after 3 washes with PBS).

We synthesized the corresponding analogues of the 101A and 101B sequences using an automated peptide synthesizer and standard Fmoc/tBu SPPS conditions (Figures S7–S8 and Supporting Information for synthetic and characterization details). In order to derivatize the peptides with the fluorogenic benzoselenadiazole amino acid, we incorporated the Fmoc‐protected amino acid **12** at the N‐terminal end of the 101A peptide to produce **5**‐**101A** (Figure 3) as the “imager peptide” and a biotin group on the 101B peptide as the “anchor peptide” (**biotin‐101B**, Figure 3). All peptides were purified by semi‐preparative HPLC and obtained in >95 % purities and suitable amounts for biological evaluation (Figure S9).

First, we assessed the influence of the benzoselenadiazole amino acid **5** on the secondary structure of the sequence 101A by performing circular dichroism (CD) spectroscopy of the peptide **5‐101A**. To run these experiments, we synthesized the N‐terminal acetylated peptide (**Ac‐101A**) as the unlabeled control (Figure S7). Notably, we observed that the secondary structure signature of **Ac‐101A** was retained in the fluorogenic **5‐101A** (Figure S10), highlighting the marginal impact of the fluorophore. The peptide **5‐101A** also maintained the environmental sensitivity of the benzoselenadiazole fluorophore, unlike the always‐on control peptide **fluorescein‐101A**, where one molecule of fluorescein was conjugated to the the N‐terminal end of the 101A sequence (Figure S11).

We corroborated that the binding of **5‐101A** to its complementary **biotin‐101B** sequence led to increased fluorescence signals, confirming the switch‐on character of the amino acid **5** and its application as a wash‐free reporter of target binding using fluorescence microscopy (Figure 3). Importantly, we also confirmed that fluorescent signals from **5‐101A** (green, Figure 3c) were due to its interaction with the **biotin‐101B** peptide by observing co‐localization between the emission of the benzoselenadiazole fluorophore and the AF647‐labeled streptavidin bound to the biotin group (red, Figure 3c). These results demonstrate the application of benzoselenadiazole amino acids as fluorogenic reporters for peptide‐guided imaging under wash‐free conditions with remarkably low background signals.

### Peptide‐PAINT Imaging of PDS‐95 Protein Nanoclusters in Mouse Brain Synaptosomes

Finally, we explored the utility of benzoselenadiazoles for peptide‐PAINT imaging of endogenous proteins in complex environments. Specifically, we synthesized peptides targeting postsynaptic density protein‐95 (PSD95), a major scaffolding protein found in postsynaptic densities in the brain. Because the protein PSD95 can form nanoclusters in native environments,^[23]^ we envisaged that PSD95‐targeting fluorogenic peptides would enable non‐invasive SR microscopy of such nanoclusters in mouse synaptosomes. PSD95 contains three PDZ domains in the postsynaptic membrane of neural excitatory synapses, which are critical for peptide binding and protein‐protein interactions.[^7f^, ^24^] Previous reports have identified the short peptides KQTSV and SSIESDV as high‐affinity binders of the PDZ domain;[^24^, ^25^] however, these sequences have not been derivatized as fluorogenic probes for direct imaging of PSD95 structures. We incorporated the benzoselenadiazole fluorophore at the N‐terminal end of the KQTSV sequence and as a replacement of isoleucine in the SSIESDV sequence to obtain the peptides **15** and **16**, respectively. Both peptides **15** and **16** were synthesized using SPPS and isolated in purities >95 % (Figures S12–S13 and Supporting Information for details).

**Figure 4:**
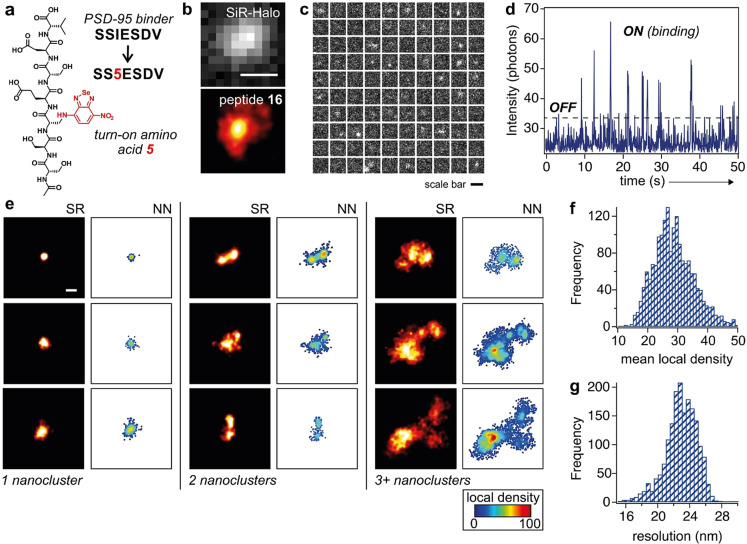
a) Chemical structure of the PSD95‐binding peptide **16** highlighting the replacement of Ile in the original sequence by amino acid **5**. b) Representative fluorescence microscopy images of PSD95‐HaloTag within a synaptosome labeled with SiR‐HaloTag ligand (40 μM, top: diffraction limited image) and peptide **16** (500 nM, bottom: peptide‐PAINT image). Scale bar: 500 nm. c) Montage of frames, starting from the top left frame and going from left to right and top to bottom with 1 second separation between frames, from time‐lapse fluorescence microscopy of a single synaptosome after incubation with peptide **16** (Movie S2 in Supporting Information). Scale bar: 500 nm. d) Longitudinal plot of fluorescence emission of peptide **16** within the synaptosome shown in c. The peaks “ON” indicate the binding of peptide **16** to the PDZ domain and the troughs “OFF” indicate the dissociation of peptide **16** from the PDZ domain. e) Representative fluorescence microscopy images from different synapse subtypes as defined by the number of PSD95 nanoclusters per postsynaptic density (PSD). The left panels display peptide‐PAINT SR microscopy images of PSD95 in synaptosomes, and the right panels display the same images after nearest neighbor (NN) analyses, which feature high local density in the protein nanoclusters. Scale bar: 500 nm. f, g) Representative histograms of the mean local density (top, determined by counting the number of neighbors within a radius of each molecule scaled to the mean density in its synaptosome) and the effective map resolution for each synaptosome (bottom).

Next, we prepared synaptosomes from brain tissues of genetically‐edited mice that expressed the endogenous protein PSD95 fused to HaloTag.[^26^, ^27^] We incubated synaptosomes with peptides **15** or **16** and a silicon rhodamine (SiR)‐HaloTag ligand, followed by imaging of the individual synapses. The binding of peptides to the PSD95 protein led to bright fluorescence signals that co‐localized with the emission from the SiR‐HaloTag ligand (Figure 4b for peptide **16** and Figure S14 for peptide **15**). Notably, the switch‐on character of peptide **16** and its transient binding to the PDZ domains enabled the visualization of PSD95 nanoclusters in the synaptosomes with a precision of 20.7±0.1 nm, a resolution of 60.8±4.8 nm, as determined by Fourier Ring Correlation,^[28]^ and S/B ratios of 14.8±1.3.

Finally, we performed peptide‐PAINT imaging to quantify the PSD95 nanoclusters in the synaptosomes of mouse brain tissue. Peptide‐PAINT images revealed hotspots with high density of localization, like those previously observed using PALM microscopy^[23a]^ (Figure 4e and Figure S14). Furthermore, we quantified the local density of the fluorescence signals from peptides **15** and **16** and visualized the distribution of individual PSD95 molecules within the synaptosomes (Figure 4e and Figure S14). This analysis resulted in high‐resolution molecular density maps of individual synaptosomes, which revealed remarkable heterogeneity in the PSD95 nanoclusters, with some synaptosomes containing only one cluster, whereas others contained two clusters or more. These results confirmed the application of benzoselenadiazole‐labeled peptides to nanoscopically image the native distribution of endogenous PSD95 in brain tissues. Unlike encoded fluorescent proteins,^[23a]^ the small size and switch‐on character of fluorogenic peptides will enable new SR imaging studies of protein localization and turnover, as well as translational approaches for the rapid detection of synaptic degradation in clinical samples from patients with neurodegenerative disorders.

## Conclusion

In the present work we report the first collection of nitrobenzodiazole amino acids as small‐sized building blocks for the construction of fluorogenic probes and peptide‐guided super‐resolution imaging. The optical characterization of the library identified the amino‐functionalized benzoselenadiazole **5** as a red‐emitting and photostable amino acid with optimal fluorogenicity. The amino acid **5** is chemically robust, scalable and compatible with most SPPS procedures, enabling the generic labeling of peptide sequences. Using different optical and biophysical methods, we have demonstrated that benzoselenadiazole‐containing peptides retain the structure and binding properties of unlabeled peptides, and their switch‐on character provides high signal‐to‐background ratios in wash‐free fluorescence microscopy. Finally, we have applied benzoselenadiazole‐labeled peptides for imaging of endogenous PSD95 nanoclusters in mouse brain samples with high localization precision. The compatibility of these small fluorogens with peptide‐PAINT imaging will create new avenues for the rational design of peptide‐based probes for minimally invasive and targeted biological imaging.

## Conflict of interest

The University of Edinburgh has filed a patent covering some of the technology described in this manuscript. The company Tocris Bioscience obtained a license to commercialize compound **1** (SCOTfluor 510, fluoro), compound **12** (SCOTfluor 510 Dapa) and the Fmoc‐protected derivative of compound **7** (SCOTfluor 470 Dapa).

1

## Supporting information

As a service to our authors and readers, this journal provides supporting information supplied by the authors. Such materials are peer reviewed and may be re‐organized for online delivery, but are not copy‐edited or typeset. Technical support issues arising from supporting information (other than missing files) should be addressed to the authors.

Supporting Information

Supporting Information

Supporting Information

## Data Availability

The data that support the findings of this study are available from the corresponding author upon reasonable request.
