# Supplementary material for: Small Fluorogenic Amino Acids for Peptide‐Guided Background‐Free Imaging
Source: Angew Chem Int Ed Engl. 2022 Dec 14;62(4):e202216231. doi: 10.1002/anie.202216231 (PMC10108274; doi:10.1002/anie.202216231)
Supplement: Supplementary file 1 — Supporting Information [file ANIE-62-0-s002.pdf]

Angewandte Chemie
Eine Zeitschrift der Gesellschaft Deutscher Chemiker

Supporting Information

Small Fluorogenic Amino Acids for Peptide-Guided Background-Free Imaging

F. de Moliner, Z. Konieczna, L. Mendive-Tapia, R. S. Saleeb, K. Morris, J. A. Gonzalez-Vera, T. Kaizuka, S. G. N. Grant, M. H. Horrocks, M. Vendrell**

Supporting Information

Table of Contents

1. Supplementary Figures
2. Supplementary Discussion
3. Materials and Methods
4. NMR Spectra
5. Supplementary References

Supplementary Figures

Dye	Abs λ (nm)	Em λ (nm)	QY	ε (M^{-1} cm^{-1})
**Cy3B[1]**	558	572	0.67	130,000
**Atto655[2]**	660	677	0.30	102,000
**Atto647N[3]**	644	669	0.65	150,000
**JF549[4]**	549	571	0.88	101,000
**AF594[5]**	590	619	0.66	93,000

Figure S1. Photophysical properties of conventional dyes for PAINT imaging. Data in aqueous solutions obtained from literature reports.

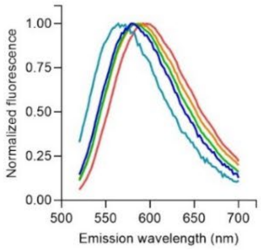

COMPOUND 5	
solvent	λ_{em} (nm)
Toluene	580
$CHCl_3$	564
THF	582
EtOH	588
DMSO	596
H_2O	606

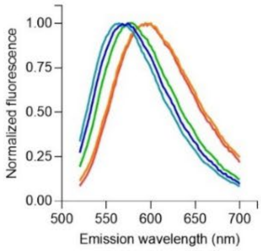

COMPOUND 6	
solvent	λ_{em} (nm)
Toluene	574
$CHCl_3$	564
THF	578
EtOH	600
DMSO	594
H_2O	606

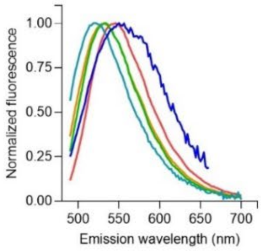

COMPOUND 7	
solvent	λ_{em} (nm)
Toluene	550
$CHCl_3$	520
THF	532
EtOH	532
DMSO	546
H_2O	548

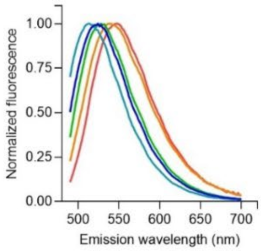

COMPOUND 8	
solvent	λ_{em} (nm)
Toluene	524
$CHCl_3$	514
THF	528
EtOH	540
DMSO	546
H_2O	556

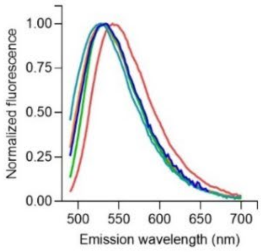

COMPOUND 9	
solvent	λ_{em} (nm)
Toluene	534
$CHCl_3$	528
THF	532
EtOH	532
DMSO	542
H_2O	546

Figure S2. Solvatochromic behavior of compounds 5-9. Amino acids (50 μM) were diluted in different solvents and their fluorescence emission intensities were recorded at 25 ^{o}C. Values presented as means (n=3). λ_{ex} = 480 nm (**5**-**6**) and 450 nm (**7**-**9**).

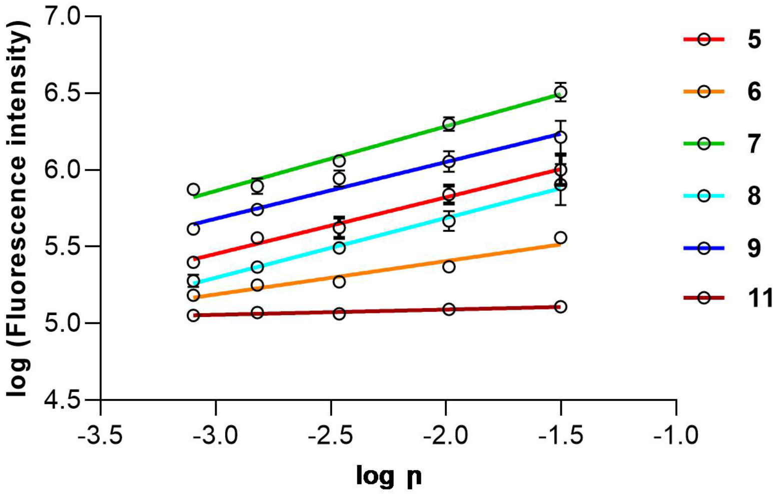

Figure S3. Viscosity sensitivity experiments. Amino acids (50 μM) were diluted with mixtures of glycerol and water and their fluorescence emission intensities were recorded at 25 °C. Values presented as means ± SD (n=6). Viscosity sensitivities were determined using the equation reported by Förster–Hoffmann.[6] Excitation wavelengths: 470 nm (for compounds **5-9**), 520 nm (for compound **11**).

**compound**	dioxane	DMSO	water
**5**	5.65 ± 0.02	3.03 ± 0.05	0.46 ± 0.01
**6**	5.15 ± 0.03	2.88 ± 0.08	0.40 ± 0.01
**7**	9.62 ± 0.09	2.61 ± 0.05	0.25 ± 0.01
**8**	9.11 ± 0.17	2.90 ± 0.03	0.50 ± 0.01

Figure S4. Fluorescence lifetimes of nitrobenzodiazole amino acids 5-8 in different solvents. Fluorescence lifetimes were obtained with λ_{ex} = 440 nm, as global fits of three fluorescence decay traces were collected at λ_{max}^{em} –10 nm, λ_{max}^{em}, and λ_{max}^{em} +10 nm. Values presented as average fluorescence lifetime of biexponential decays, and the errors associated to the average fluorescence decay time are the standard deviations of the values obtained at the three different emission wavelengths.

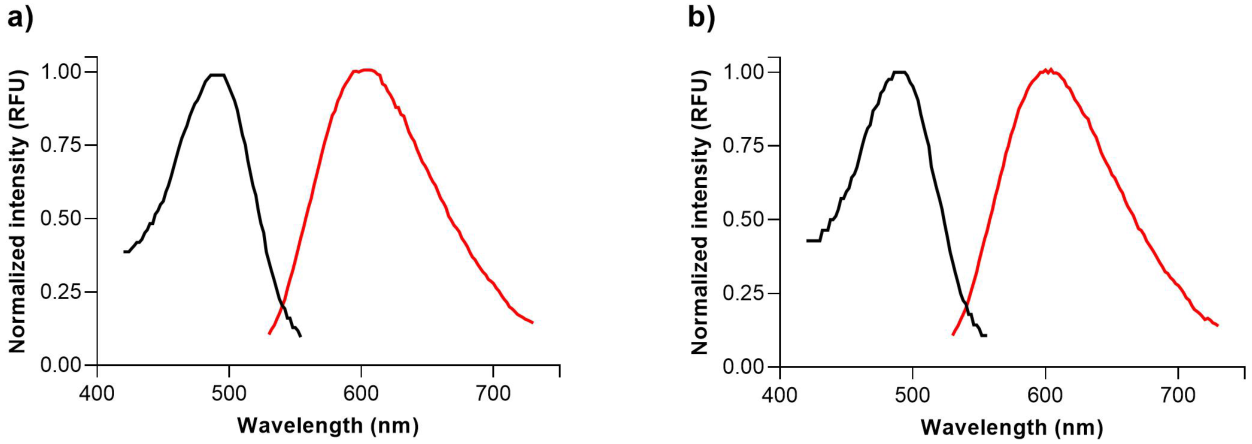

Figure S5. Absorbance (black) and fluorescence emission (red) spectra of compounds **13** and **14** in DMSO (both at 10 μM, excitation: 500 nm).

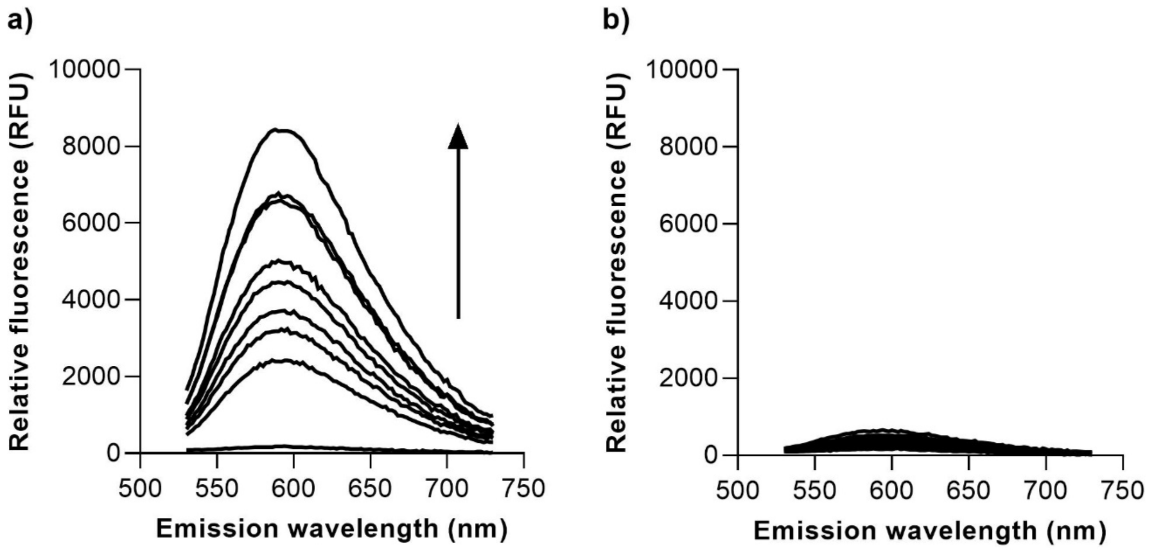

Figure S6. Fluorescence emission spectra of compounds **13** (a) and **14** (b) (both at 10 μM) in PBS suspensions with increasing concentrations of phosphatidylcholine liposomes (excitation: 500 nm).

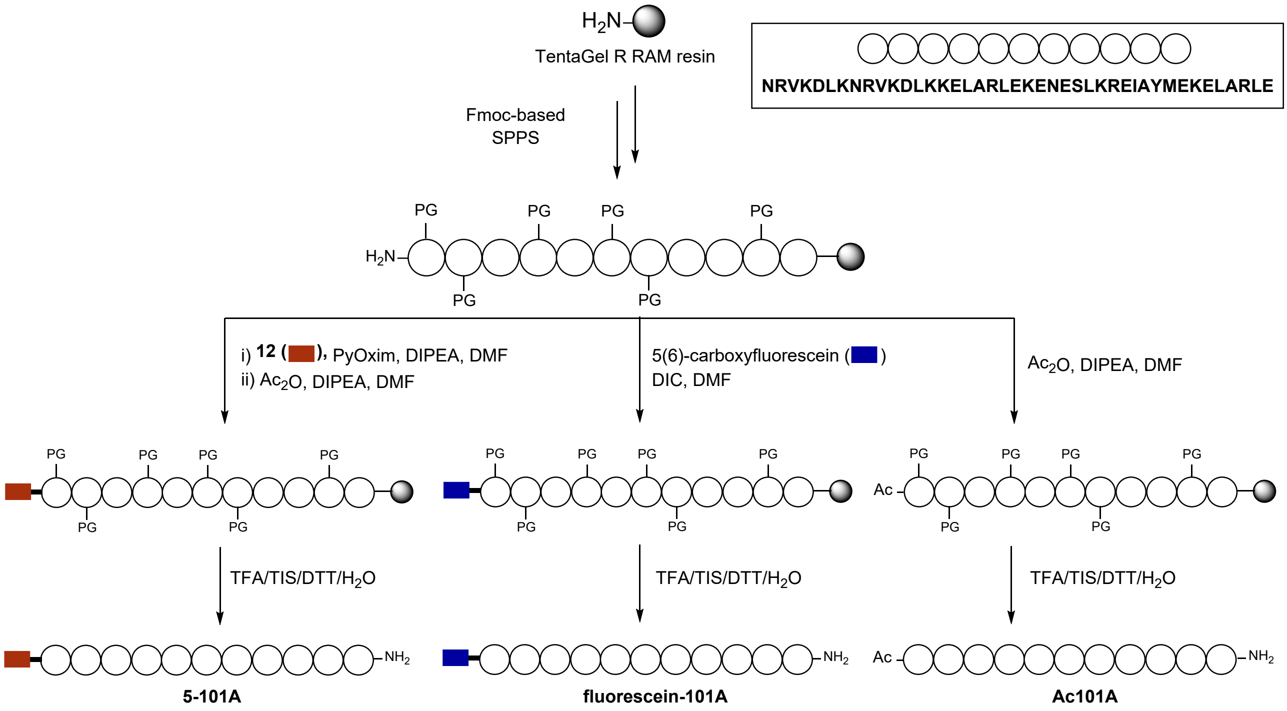

Figure S7. Synthetic scheme for the preparation of 101A analogues (**Ac-101A**, **5-101A** and **fluorescein-101A**).

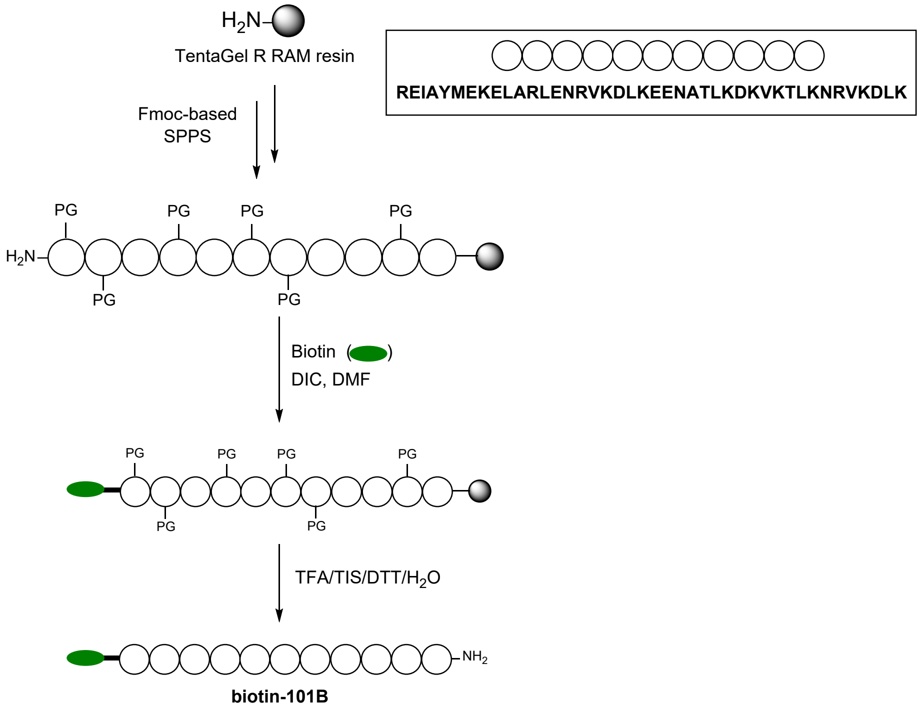

Figure S8. Synthetic scheme for the preparation of **biotin-101B**.

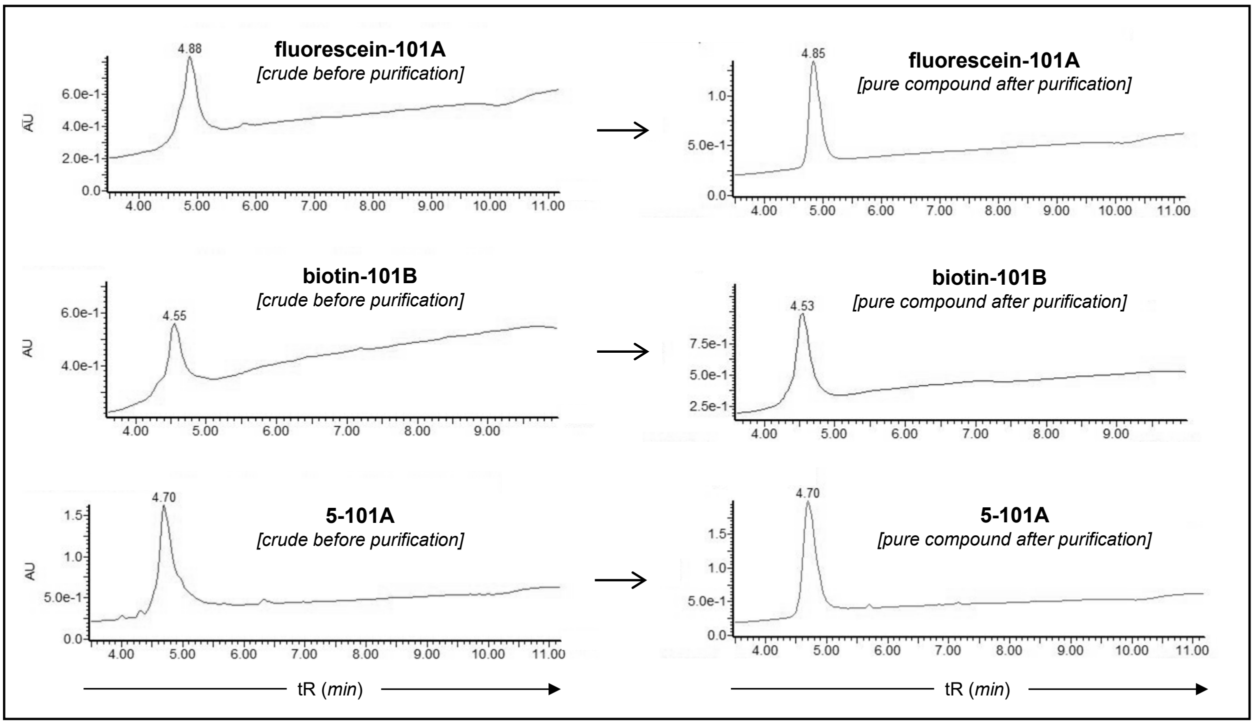

Figure S9. HPLC traces of peptide analogues for the 101A and 101B sequences. UV detection at 220 nm.

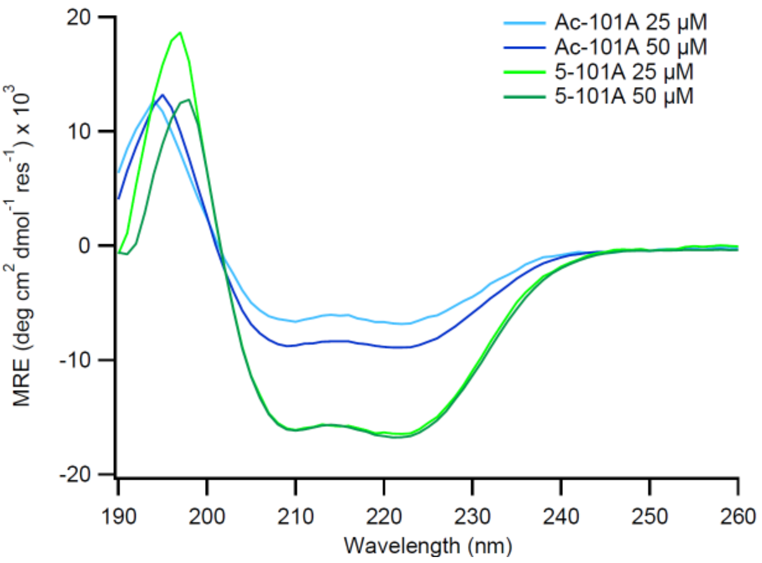

Figure S10. Circular dichroism traces for compounds **Ac-101A** and **5-101A**. Both peptides exhibit a double dip (210 nm and 225 nm) characteristic of alpha helices, confirming that the incorporation of amino acid **5** does not interfere with the secondary structure of the 101A peptide.

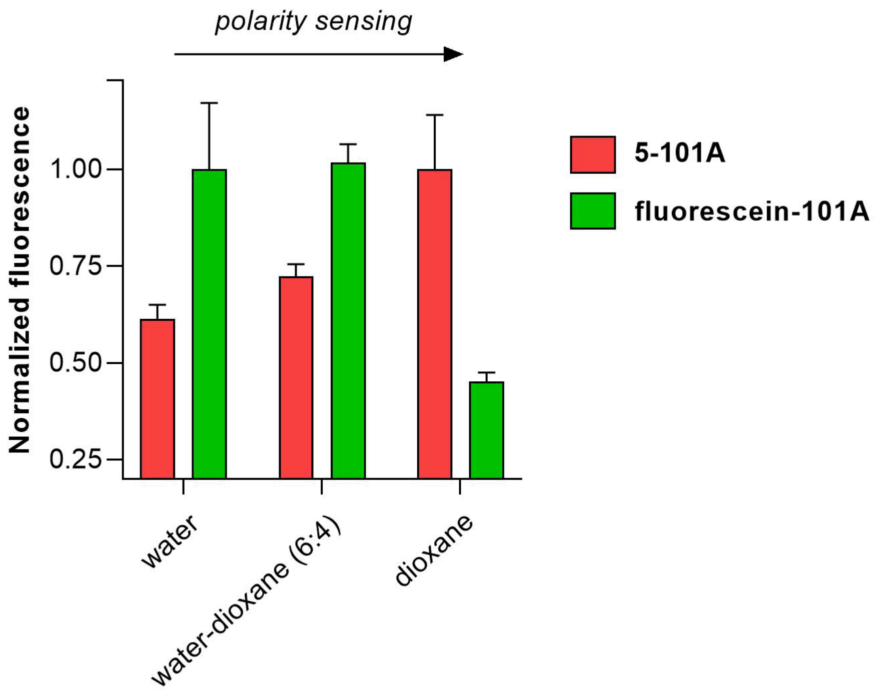

Figure S11. Fluorescence emission of the peptides **5-101A** and **fluorescein-101A** (both at 10 μM) in different mixtures of water and dioxane (**5-101A**: exc 500 nm, em 600 nm; **fluorescein-101A**: exc 450 nm, em 500 nm).

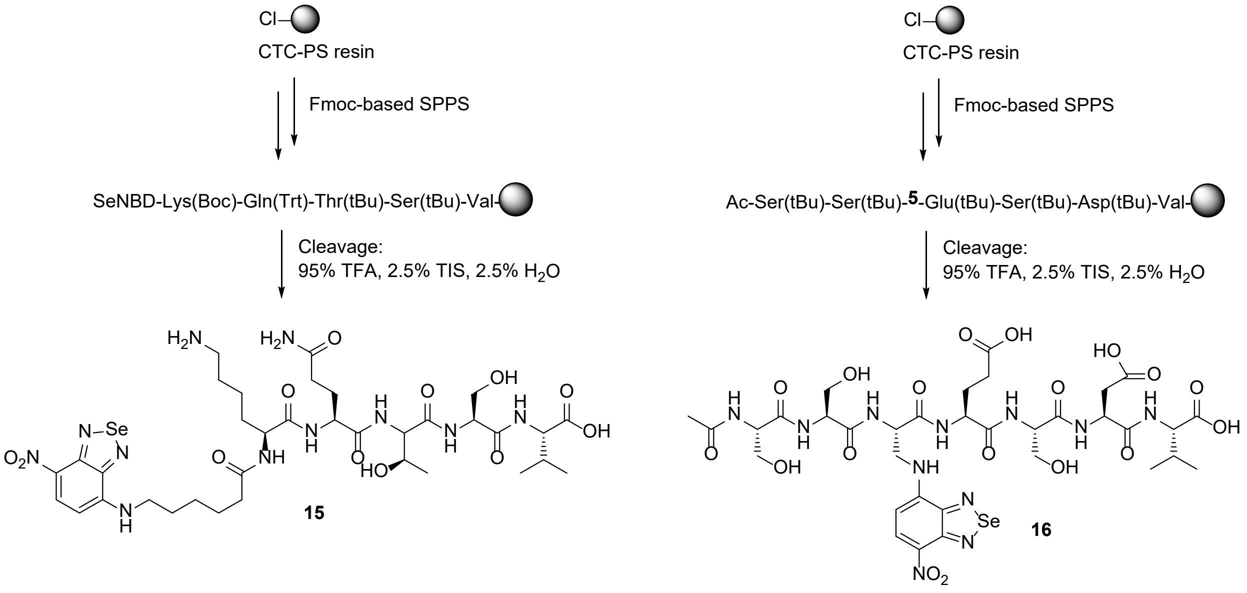

Figure S12. Synthetic scheme for the preparation of peptides **15** and **16**.

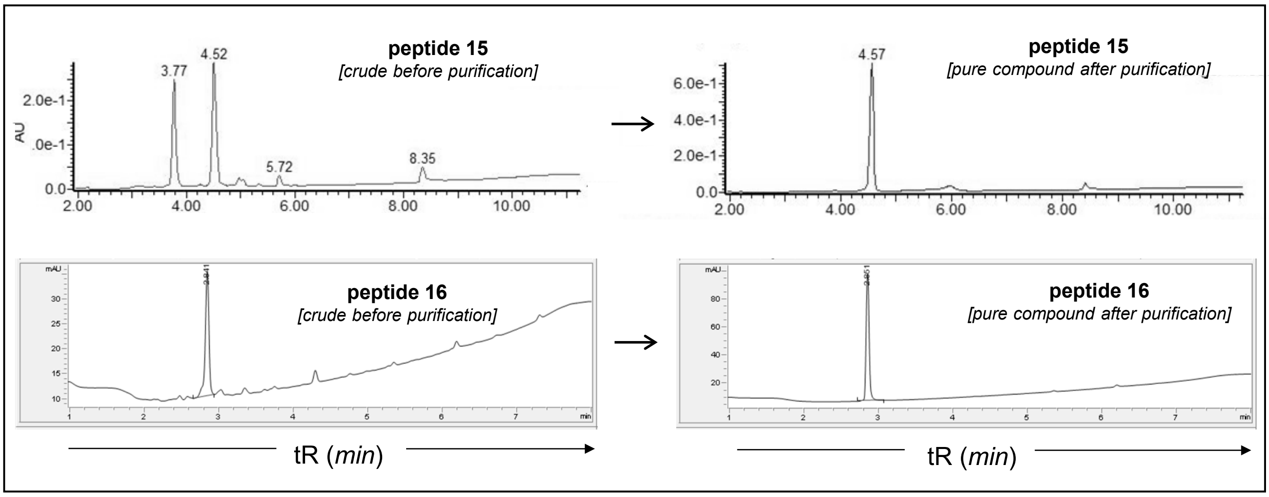

Figure S13. HPLC traces for crude mixtures and purified peptides **15** and **16**. UV detection at 254 nm. For peptide **15**, an additional side peak with maximum absorbance at 330 nm instead of 488 nm (amino acid **5**) was also detected.

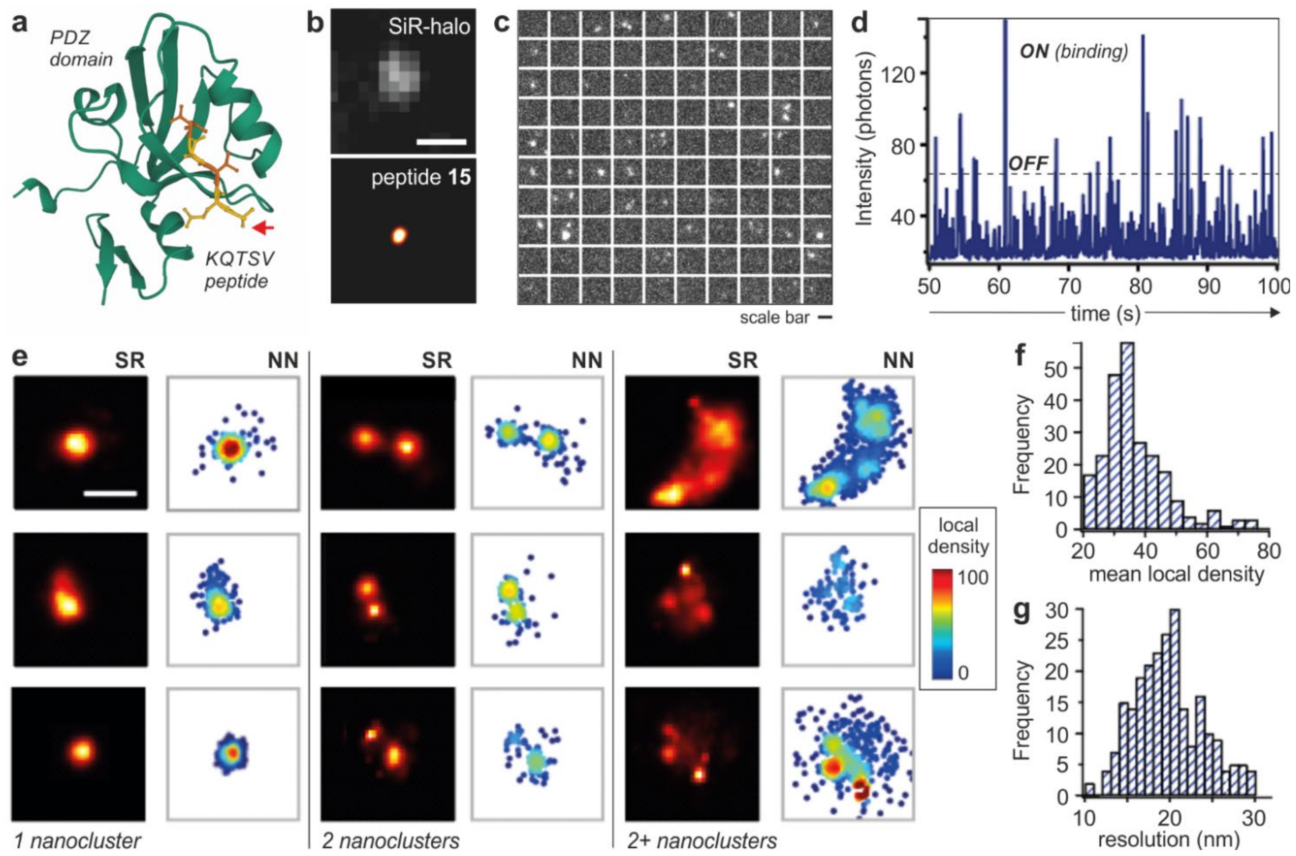

Figure S14. PAINT imaging of synaptosomes with peptide 15. a) Schematic illustration of the binding between the KQTSV peptide (yellow) and the third PDZ domain of PSD95 in synaptosomes (green, Protein Data Bank code reference 1BE9). The red arrow points at the N-terminal end of KQTSV. b) Representative fluorescence microscopy images of PSD95-HaloTag within a synaptosome labeled with SiR-HaloTag ligand (40 μM, top: diffraction limited image) and peptide **15** (500 nM, bottom: peptide-PAINT image). Scale bar: 500 nm. c) Montage of frames from time-lapse fluorescence microscopy of a single synaptosome after incubation with peptide **15** (Movie S1). Scale bar: 1.5 μm. d) Longitudinal plot of fluorescence emission of peptide **15** within the synaptosome shown in c. The peaks 'ON' indicate the binding of peptide **15** to the PDZ domain and the troughs 'OFF' indicate the dissociation of peptide **15** from the PDZ domain. e) Representative fluorescence microscopy images from different synapse subtypes as defined by the number of PSD95 nanoclusters per postsynaptic density (PSD). The left panels display peptide-PAINT SR microscopy images of PSD95 in synaptosomes, and the right panels display the same

images after nearest neighbor (NN) analyses, which feature high local density in the protein nanoclusters. Scale bar: 200 nm. f, g) Representative histograms of the mean local density (top, determined by counting the number of neighbors within a radius of each molecule scaled to the mean density in its synaptosome) and the effective map resolution for each synaptosome (bottom).

Movie Legends

Movie S1. Video showing the fluorescence switching of peptide **15** as it binds and unbinds the protein PSD-95 in an individual synaptosome.

Movie S2. Video showing the fluorescence switching of peptide **16** as it binds and unbinds the protein PSD-95 in an individual synaptosome.

Supplementary Discussion

Considerations about the implementation of the building block 5 in SPPS.

As exemplified by the synthesis of peptide **16**, the amino acid **5** proves to be fully stable for several coupling and deprotection cycles (at least 3 cycles). The stability was confirmed by HPLC analysis of the peptide crude, where no significant side products were detected (see Supplementary Figure S13). Similar trends were observed during the synthesis of peptide **5-101A** (see Supplementary Figure S9). These results confirm that the building block **5** is stable after multiple couplings in longer peptide sequences without causing any major stability issues. Furthermore, the amino acid **5** withstands treatment with 95% TFA for several hours at r.t. in the presence of common scavengers (e.g., TIS, DTT and H_2O).

Materials and Methods

General materials.

Commercially available reagents were used without further purification. Thin-layer chromatography was conducted on Merck silica gel 60 F254 sheets and visualized by UV (254 and 365 nm). Silica gel (particle size 35–70 µm) was used for column chromatography. ^{1}H and ^{13}C spectra were recorded in a Bruker Avance 500 spectrometer (at 500 and 125 MHz, respectively). Data for ^{1}H NMR spectra are reported as chemical shift δ (ppm), multiplicity, coupling constant (Hz) and integration. Data for ^{13}C NMR spectra reported as chemical shifts relative to the solvent peak. HPLC-MS analysis was performed on a Waters Alliance 2695 separation module connected to a Waters PDA2996 photodiode array detector and a ZQ Micromass mass spectrometer (ESI-MS) with a Phenomenex® column (C_{18}, 5 µm, 4.6 × 150 mm). Peptide purifications were conducted in a semi-Preparative Agilent HPLC consisting of a 1220 Infinity II autosampler and a 1260 Infinity II detector. Kinetex 150 × 21.2 mm (5 µm) C18 column was used, together with H_2O (0.1% HCOOH) and CH_3CN (0.1% HCOOH) as eluents and a flow rate of 8 mL min^{-1}. HRMS (ESI positive) were obtained with a Bruker ESI Micro-TOF mass spectrometer. MALDI analysis was performed on a Bruker UltrafleXtreme MALDI TOF-TOF mass spectrometer.

Chemical synthesis.

General strategy for the preparation of amino acids 5–10.

A solution of Boc-L-amino acid (1.1 eq) and $NaHCO_3$ (3 eq) in water was heated at 55 ºC. A solution of 4-fluoro(chloro)-7-nitrobenzo[c][1,2,5]diazole (**1**, **2** or **3**, 1 eq)[7] in MeOH was then added dropwise and the reaction was kept stirring at 55 ºC until completion (by TLC and HPLC). The solvents were removed under reduced pressure and the crude product was quickly purified by column chromatography (DCM:MeOH) to give the N-Boc-(*R*)-2-amino(thio)-3-((7-nitrobenzo[c][1,2,5]diazol-4-yl)amino)propanoic acid hydrochloride intermediate as a red or orange solid, that was redissolved in 4 N HCl in dioxane and stirred for 30 min at r.t. to give the expected amino acid upon removal of the solvent. This procedure is compatible with the synthesis of amino acids up to ~100 mg scale.

(*S*)-2-amino-3-((7-nitrobenzo[c][1,2,5]selenadiazol-4-yl)amino) propanoic acid hydrochloride (5)

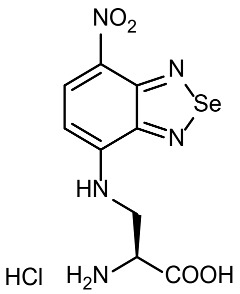

Obtained from Boc-L-2,3-diaminopropionic acid (42 mg, 0.21 mmol, 1.1 eq) and 4-fluoro-7-nitrobenzo[c][1,2,5] selenadiazole **1** (46 mg, 0.19 mmol, 1 eq) upon 30 min reaction at 55 ºC and purification of the intermediate with (DCM:MeOH 8:2). Deprotection with 4 N HCl in dioxane gave compound **5** (82 mg, red solid, quantitative yield over two steps).

^{1}H NMR (500 MHz, DMSO-*d_6*) δ 8.60 (d, *J* = 8.8 Hz, 1H), 8.50 (br, s, 2H), 8.27 (s, 1H), 6.56 (d, *J* = 8.9 Hz, 1H), 4.20 (t, *J* = 5.9 Hz, 1H), 4.10 – 3.88 (m, 2H).

^{13}C NMR (125 MHz, DMSO-*d_6*) δ 169.6, 152.5, 152.2, 148.2, 135.2, 129.7, 98.5, 51.7.

HRMS (m/z, ESI): calcd for $C_9H_8N_5O_4Se$ $[M - H]^-$: 329.9747, found: 329.9760.

(*R*)-2-amino-3-((7-nitrobenzo[c][1,2,5]selenadiazol-4-yl)mercapto) propanoic acid hydrochloride (6)

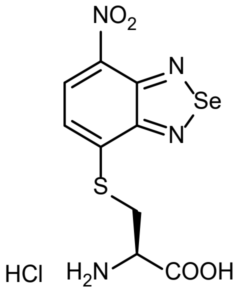

Obtained from Boc-L-cysteine (20 mg, 0.09 mmol, 1.1 eq) and 4-fluoro-7-nitrobenzo[c][1,2,5] selenadiazole **1** (20 mg, 0.08 mmol, 1 eq) upon 30 min reaction at 55 °C and purification of the intermediate with (DCM:MeOH 8:2). Deprotection with 4 N HCl in dioxane gave compound **6** (25 mg, orange solid, 80% over two steps).

^{1}H NMR (500 MHz, MeOD) δ 8.49 (d, *J* = 7.9 Hz, 1H), 7.65 (d, *J* = 8.0 Hz, 1H), 4.43 (dd, *J* = 7.7, 4.2 Hz, 1H), 4.01 (dd, *J* = 14.8, 4.2 Hz, 1H), 3.79 (dd, *J* = 14.8, 7.8 Hz, 1H).

^{13}C NMR (125 MHz, MeOD) δ 158.3, 150.5, 139.8, 139.0, 127.4, 122.0, 51.7, 31.1, 29.3.

HRMS (m/z, ESI): calcd for $C_9H_9N_4O_4SSe$ $[M+H]^+$: 348.9509, found: 348.9504.

(*S*)-2-amino-3-((7-nitrobenzo[c][1,2,5]thiadiazol-4-yl)amino) propanoic acid hydrochloride (7)

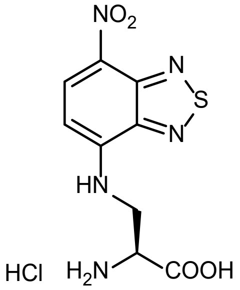

Obtained from Boc-L-2,3-diaminopropionic acid (35 mg, 0.21 mmol, 1.1 eq) and 4-fluoro-7-nitrobenzo[c][1,2,5] thiadiazole **2** (30 mg, 0.15 mmol, 1 eq) upon 15 min reaction at 55 °C

and purification of the intermediate with (DCM:MeOH 8:2). Deprotection with 4 N HCl in dioxane gave compound **7** (52 mg, yellow solid, quantitative yield over two steps).

^{1}H NMR (500 MHz, DMSO-*d_6*) δ 8.67 (d, *J* = 8.9 Hz, 1H), 8.51 (s, 1H), 8.40 (br, s, 2H), 6.72 (d, *J* = 9.0 Hz, 1H), 4.26 (t, *J* = 6.1 Hz, 1H), 4.01 – 3.91 (m, 2H).

^{13}C NMR (125 MHz, DMSO-*d_6*) δ 169.5, 148.3, 147.2, 147.0, 134.4, 127.8, 100.6, 51.9, 43.3.

HRMS (m/z, ESI): calcd for $C_9H_{10}N_5O_4S$ $[M+H]^+$: 284.0453, found: 284.0453.

(*R*)-2-amino-3-((7-nitrobenzo[c][1,2,5]thiadiazol-4-yl)mercapto) propanoic acid hydrochloride (8)

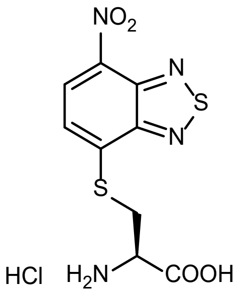

Obtained from Boc-*L*-cysteine (24 mg, 0.11 mmol, 1.1 eq) and 4-fluoro-7-nitrobenzo[c][1,2,5] thiadiazole **2** (20 mg, 0.10 mmol, 1 eq) upon 20 min reaction at 55 ºC and purification of the intermediate with (DCM:MeOH 85:15 → 75:25). Deprotection with 4 N HCl in dioxane gave compound **8** (34 mg, yellow solid, quantitative yield over two steps).

^{1}H NMR (500 MHz, MeOD) δ 8.63 (d, *J* = 8.1 Hz, 1H), 7.81 (d, *J* = 8.1 Hz, 1H), 4.41 (dd, *J* = 7.7, 4.2 Hz, 1H), 4.07 (dd, *J* = 14.8, 4.2 Hz, 1H), 3.84 (dd, *J* = 14.9, 7.7 Hz, 1H).

^{13}C NMR (125 MHz, MeOD) δ 168.6, 153.8, 146.1, 138.4, 137.4, 127.5, 123.1, 51.7, 31.2.

HRMS (m/z, ESI): calcd for $C_9H_9N_4O_4S_2$ $[M+H]^+$: 301.0060, found: 301.0061.

(*S*)-2-amino-3-((7-nitrobenzo[c][1,2,5]oxadiazol-4-yl)amino) propanoic acid hydrochloride (9)

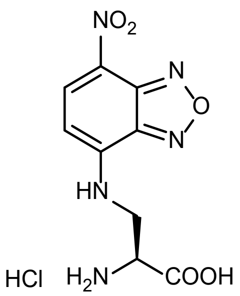

Obtained from Boc-*L*-2,3-diaminopropionic acid (22 mg, 0.11 mmol, 1.1 eq) and 4-chloro-7-nitrobenzo[c][1,2,5] oxadiazole **3** (20 mg, 0.10 mmol, 1 eq) upon 20 min reaction at 55 ºC and purification of the intermediate with (DCM:MeOH 85:15 → 75:25). Deprotection with 4 N HCl in dioxane gave compound **9** (26 mg, orange solid, 86% over two steps).

^{1}H NMR (500 MHz, DMSO-d_6) δ 8.57 (d, *J* = 8.9 Hz, 1H), 6.50 (d, *J* = 8.9 Hz, 1H), 4.07 – 3.79 (m, 3H).

^{13}C NMR (125 MHz, DMSO-d_6) δ 163.6, 147.8, 144.6, 138.5, 124.7, 103.2, 72.6, 71.0, 60.7.

HRMS (m/z, ESI): calcd for $C_9H_{10}N_5O_5$ $[M+H]^+$: 268.0676, found: 268.0671.

(*R*)-2-amino-3-((7-nitrobenzo[c][1,2,5]oxadiazol-4-yl)mercapto) propanoic acid hydrochloride (10)

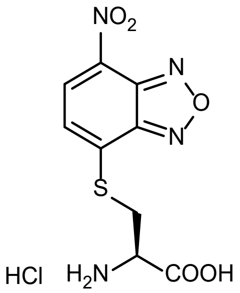

Obtained from Boc-L-cysteine (17 mg, 0.08 mmol, 1.1 eq) and 4-chloro-7-nitrobenzo[c][1,2,5] oxadiazole **3** (20 mg, 0.07 mmol, 1 eq) upon 15 mins reaction at 55 ºC and purification of the intermediate with (DCM:MeOH 85:15 → 8:2). Deprotection with 4 N HCl in dioxane gave compound **10** (24 mg, yellow solid, quantitative yield over two steps).

^{1}H NMR (500 MHz, D_2O) δ (d, *J* = 7.9 Hz, 1H), 7.58 (d, *J* = 7.9 Hz, 1H), 4.32 (dd, *J* = 7.5, 4.3 Hz, 1H), 3.99 (dd, *J* = 15.1, 4.3 Hz, 1H), 3.83 (dd, *J* = 15.1, 7.5 Hz, 1H).

^{13}C NMR (125 MHz, D_2O) δ 170.3, 149.7, 142.7, 136.5, 133.7, 132.4, 125.4, 52.2, 31.3.

HRMS (m/z, ESI): calcd for $C_9H_9N_4O_5S$ $[M+H]^+$: 285.0288, found: 285.0282.

(*S*)-2-((((9H-fluoren-9-yl)methoxy)carbonyl)amino)-3-((2,2-dimethyl-7-nitro-2H-benzo[d]imidazol-4-yl)amino)propanoic acid (11)

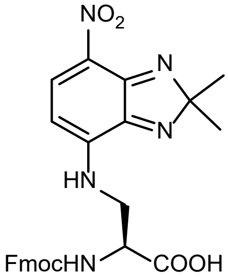

A solution of Fmoc-L-2,3-diaminopropionic acid (72 mg, 0.22 mmol, 1.1 eq) and $NaHCO_3$ (50 mg, 0.60 mmol, 3 eq) in water (5 mL) was added dropwise to a solution of 4-fluoro-2,2-dimethyl-7-nitro-2H-benzo[d]imidazole **4** (42 mg, 0.20 mmol, 1 eq) in MeCN (5 mL) and the reaction was stirred at r.t. for 6 days. The solvents were removed under reduced pressure and the crude product was purified by column chromatography (DCM:MeOH 97:3 → 95:5) to give compound **11** (25 mg, purple solid, 24%).

^{1}H NMR (500 MHz, MeOD) δ 8.43 (d, *J* = 8.3 Hz, 1H), 7.77 (d, *J* = 7.5 Hz, 2H), 7.60 (dd, *J* = 10.7, 7.8 Hz, 2H), 7.36 (t, *J* = 7.4 Hz, 2H), 7.26 (dd, *J* = 11.8, 7.3 Hz, 2H), 6.03 (d, *J* = 8.9 Hz, 1H), 4.44 – 4.37 (m, 2H), 4.28 – 4.22 (m, 1H), 4.17 (t, *J* = 6.6 Hz, 1H), 3.95 – 3.85 (m, 1H), 3.75 (dd, *J* = 13.4, 7.5 Hz, 1H), 1.52 (s, 3H), 1.48 (s, 3H).

^{13}C NMR (125 MHz, MeOD) δ 174.8, 157.1, 143.8, 143.7, 141.2, 127.4, 126.7, 124.8, 124.7, 119.5, 108.8, 95.9, 66.6, 55.1, 29.4, 21.0.

HRMS (m/z, ESI): calcd for $C_{27}H_{26}N_5O_6^+$ $[M+H]^+$: 516.1877, found: 516.1867.

(*S*)-2-((((9H-fluoren-9-yl)methoxy)carbonyl)amino)-3-((7-nitrobenzo[c][1,2,5] selenadiazol-4-yl)amino) propanoic acid (12)

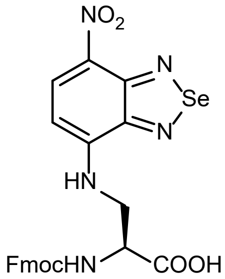

A solution of sodium azide (19 mg, 0.28 mmol, 1.2 eq) in water (5 mL) was added to a solution of 9-fluorenylmethoxycarbonyl chloride (60 mg, 0.23 mmol, 1 eq) in 1,4-dioxane (5 mL) and the mixture was stirred at r.t. for 1 h. This mixture was then added dropwise to a solution of (*S*)-2-amino-3-((7-nitrobenzo[c][1,2,5]selenadiazol-4-yl)amino) propanoic acid hydrochloride **5** (85 mg, 0.23 mmol, 1 eq) in a mixture of 15 mL of 1,4-dioxane and 15 mL of 1% aqueous $NaHCO_3$ solution. The reaction was stirred overnight at r.t., until completion (by TLC and HPLC-MS). The solvents were removed under reduced pressure and the crude product was purified by column chromatography (DCM:MeOH 8:2) to give **12** (95 mg, orange solid, 75%).

^{1}H NMR (500 MHz, DMSO-*d*$_6$) 8.62 – 8.45 (m, 1H), 8.11(br, s, 1H), 7.94 – 7.81 (m, 2H), 7.71 – 7.55 (m, 2H), 7.45 – 7.17 (m, 5H), 6.36 (d, *J* = 9.3 Hz, 1H), 4.39 – 4.10 (m, 3H), 4.10 – 3.90 (m, 1H), 3.58 – 3.48 (m, 1H), 3.46 – 3.36 (m, 1H).

^{13}C NMR (125 MHz, DMSO-*d*$_6$) δ 170.8, 156.8, 152.4, 148.1, 144.3, 141.1, 136.0, 129.4, 128.6, 128.3, 128.0, 127.8, 127.5, 127.4, 125.6, 125.5, 121.9, 120.5, 97.9, 66.0, 58.1, 47.1, 43.6.

HRMS (m/z, ESI): calcd for $C_{24}H_{19}N_5O_6SeNa$ $[M+Na]^+$: 576.0393, found: 576.0380.

(*S*)-2-(((*tert*-butoxy)carbonyl)amino)-*N*-dodecyl-3-((7-nitrobenzo [c][1,2,5]selenadiazol-4-yl)amino)propanamide (13)

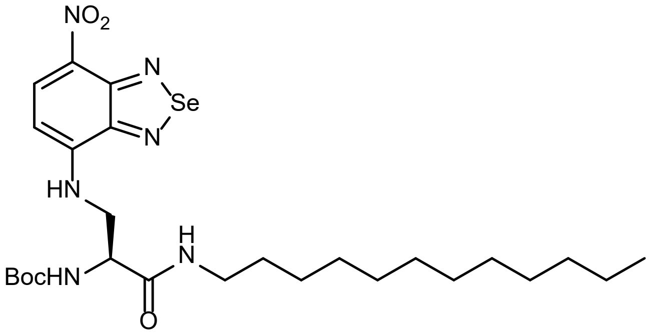

(*S*)-2-(((*tert*-butoxy)carbonyl)amino)-3-((7-nitrobenzo[c][1,2,5]selenadiazol-4-yl)amino) propanoic acid **Boc-5** (28 mg, 0.06 mmol, 1 eq) was dissolved in DMF (1.5 mL), then COMU (34 mg, 0.07 mmol, 1.2 eq) was added and the mixture was stirred at r.t. for 5 min. Dodecylamine (24 mg, 0.12 mmol, 2 eq) and DIPEA (23 µL, 0.12 mmol, 2 eq) were then added and reaction was stirred at r.t. for 1 h. The solvents were removed under reduced pressure and the crude product was purified by column chromatography (DCM:MeOH 96:4) to give compound **13** (26 mg, red solid, 67%).

^{1}H NMR (500 MHz, $CDCl_3$) δ 8.59 (d, *J* = 8.7 Hz, 1H), 7.25 (br, s, 1H), 6.45 (t, *J* = 5.2 Hz, 1H), 6.31 (d, *J* = 8.8 Hz, 1H), 5.50 (br, s, 1H), 4.55 (br, s, 1H), 3.86 – 3.72 (m, 2H), 3.39 – 3.25 (m, 2H), 1.57 – 1.51 (m, 2H), 1.49 (s, 9H), 1.35 – 1.23 (m, 18H), 0.90 (t, *J* = 7.0 Hz, 3H).

^{13}C NMR (125 MHz, $CDCl_3$) δ 169.5, 156.1, 152.4, 152.3, 147.2, 135.2, 130.2, 97.5, 81.1, 53.0, 45.8, 39.9, 31.9, 29.64, 29.62, 29.58, 29.52, 29.4, 29.3, 29.2, 28.3, 26.85, 22.7, 14.1.

HRMS (m/z, ESI): calcd for $C_{26}H_{42}N_6O_5SeNa^+$ $[M+Na]^+$: 621.2280, found: 621.2283.

(*S*)-2-(((*tert*-butoxy)carbonyl)amino)-((7-nitrobenzo[c][1,2,5]selenadiazol-4-yl)amino)-*N*-octadecylpropanamide (14)

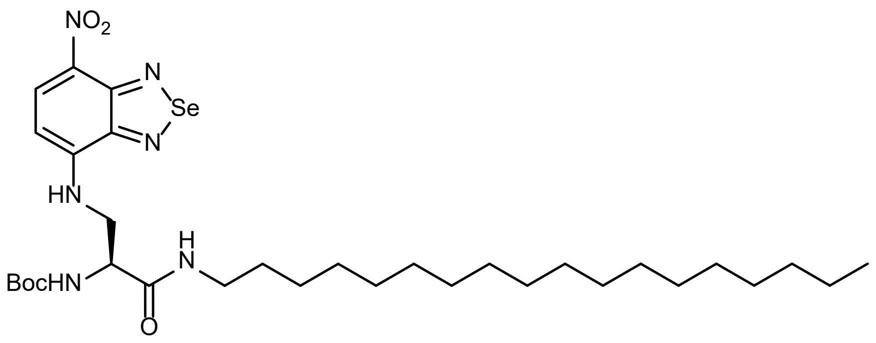

(*S*)-2-(((*tert*-butoxy)carbonyl)amino)-3-((7-nitrobenzo[c][1,2,5]selenadiazol-4-yl)amino) propanoic acid **Boc-5** (28 mg, 0.06 mmol, 1 eq) was dissolved in DMF (1.5 mL), then COMU (34 mg, 0.07 mmol, 1.2 eq) was added and the mixture was stirred at r.t. for 5 min. Octadecylamine (35 mg, 0.12 mmol, 2 eq) and DIPEA (23 µL, 0.12 mmol, 2 eq) were then added and reaction was stirred at r.t. for 1 h. The solvents were removed under reduced pressure and the crude product was purified by column chromatography (DCM:MeOH 98:2) to give compound **14** (31 mg, red solid, 70%).

^{1}H NMR (500 MHz, $CDCl_3$) δ 8.60 (d, *J* = 8.7 Hz, 1H), 7.24 (br, s, 1H), 6.41 (br, s, 1H), 6.32 (d, *J* = 8.7 Hz, 1H), 5.46 (br, s, 1H), 4.55 (br, s, 1H), 3.86 – 3.71 (m, 2H), 3.39 – 3.25 (m, 2H), 1.56 – 1.51 (m, 2H), 1.49 (s, 9H), 1.35 – 1.23 (m, 30H), 0.90 (t, *J* = 7.0 Hz, 3H).

^{13}C NMR (125 MHz, $CDCl_3$) δ 169.5, 155.9, 152.4, 152.3, 147.2, 135.2, 130.3, 97.5, 81.1, 52.9, 45.8, 39.9, 31.9, 29.70, 29.68, 29.66, 29.59, 29.53, 29.40, 29.36, 29.25, 28.3, 26.9, 22.7, 14.1.

HRMS (m/z, ESI): calcd for $C_{32}H_{54}N_6O_5SeNa^+$ $[M+Na]^+$: 705.3213, found: 705.3208.

Stability tests of compounds 5–11 under SPPS conditions. Compounds **5–11** (10 mM DMSO stock) were diluted with different reagent mixtures (final volume 500 uL) and stirred at r.t.. Conditions tested: a) TFA:DCM (95:5); b) piperidine:DMF (2:8); c) TBAF 3 H_2O (3 eq) in DCM; d) $Pd(PPh_3)_4$ (0.25 eq) in DCM; e) 10% N_2H_4 H_2O in DMF. The solutions were stirred at r.t. for 1 h (conditions a, c and e) or 30 min (conditions b and d); then, mixtures were analyzed by HPLC-MS. Representative chromatograms for compounds **5** and **6** are depicted below.

HPLC traces for compound **5** under different SPPS conditions

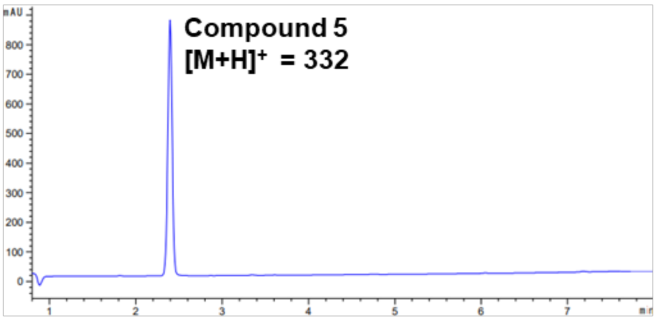

a) TFA:DCM (95:5)
purity >95%

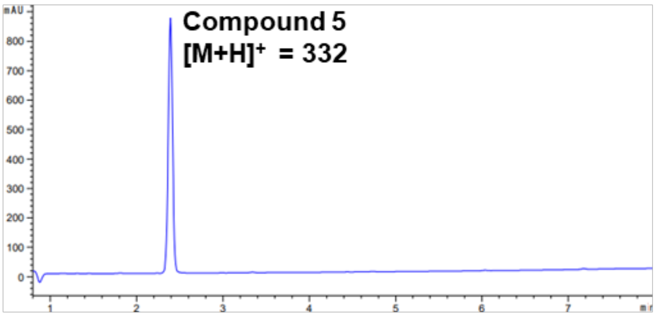

b) piperidine:DMF (2:8)
purity >95%

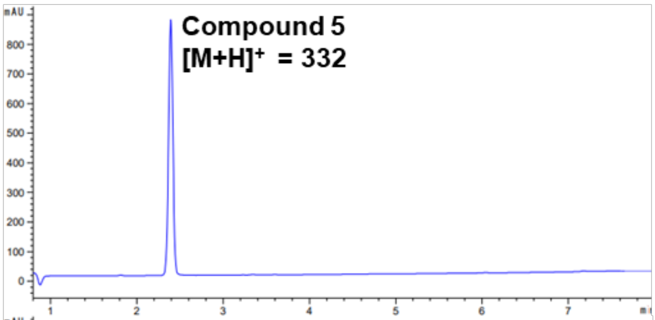

c) TBAF 3 H_2O in DCM
purity >95%

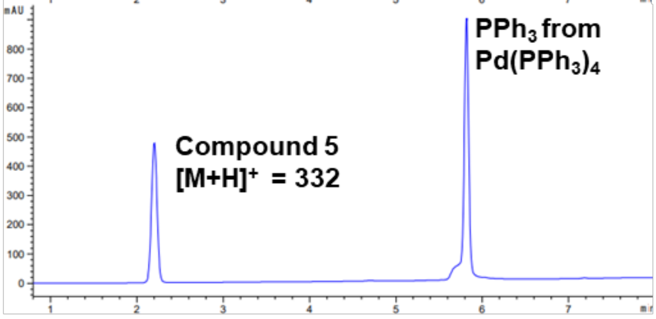

d) $Pd(PPh_3)_4$ in DCM
purity >95%

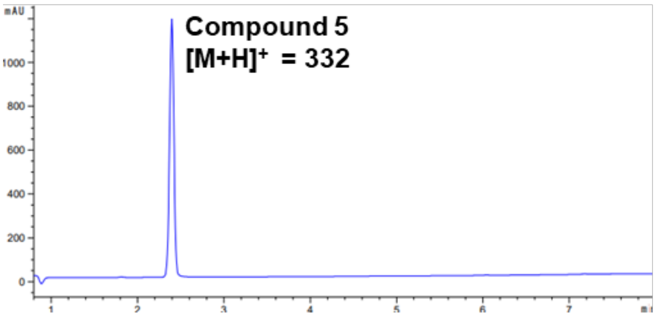

e) 10% N_2H_4 H_2O in DMF
purity >95%

HPLC traces for compound **6** under different SPPS conditions

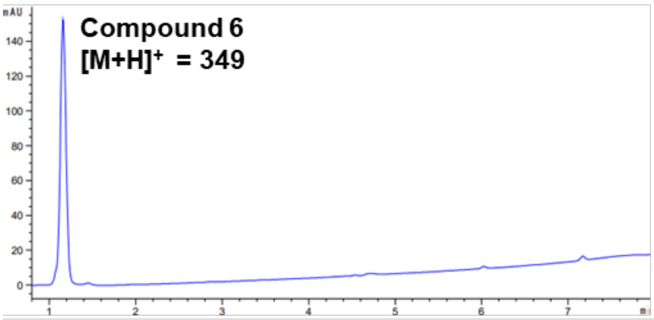

a) TFA:DCM (95:5)
purity >95%

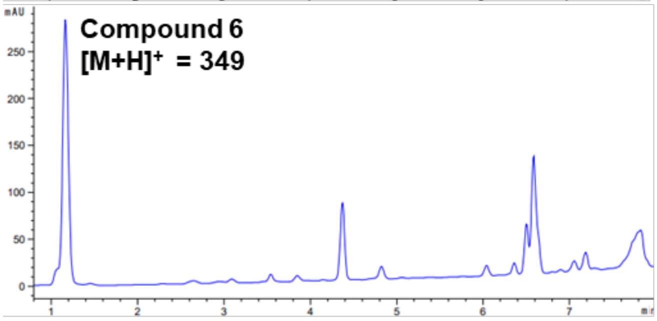

b) piperidine:DMF (2:8)
purity = 51%

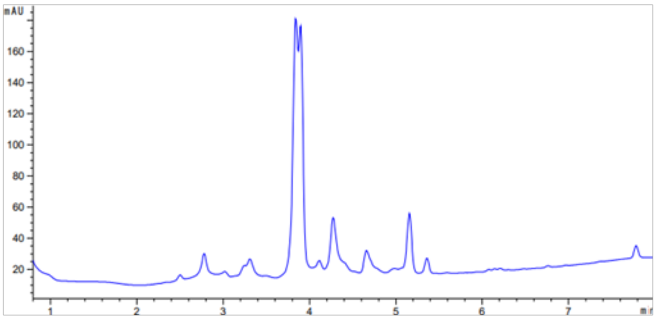

c) TBAF 3 H_2O in DCM
purity <5%

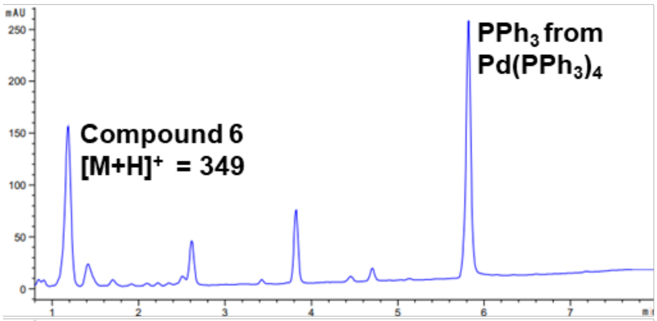

d) $Pd(PPh_3)_4$ in DCM
purity = 47%

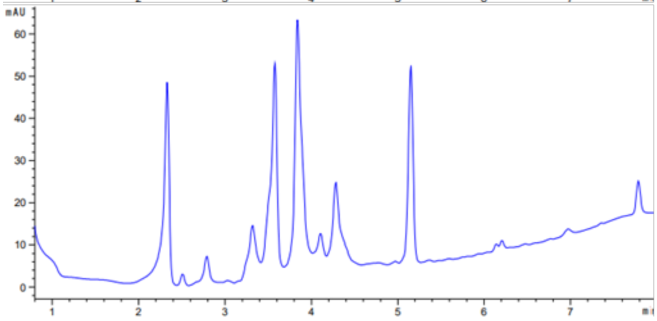

e) 10% N_2H_4 H_2O in DMF
purity <5%

General procedures for SPPS. Peptide elongation: automated microwave-assisted SPPS was carried out in a Liberty Blue microwave peptide synthesizer (CEM). DIC and OxymaPure reagents were used for each amide coupling and 5% (w/v) piperazine (0.1 M OxymaPure) in DMF was employed for the removal of Fmoc protecting groups. The amino acid **12** was manually incorporated using 5-mL polystyrene syringes fitted with polyethylene porous discs under standard manual Fmoc/tBu-SPPS at r.t. Coupling was carried out using Fmoc-AA-OH (1.2 eq.), Pyoxim (1.2 eq.), OxymaPure (1.2 eq.) and DIPEA (2.4 eq.) in DMF. Solvents, excess of reagents and soluble byproducts were removed by suction. N-terminal acetylation was performed with acetic anhydride (10 eq), DIPEA (10 eq) in DMF for 30 min at r.t. Cleavage from resin: Peptides were then cleaved from the resin with 95% TFA, 2.5% TIS, 2.5% H_2O for 1 h (peptide **15** and **16**) or 95% TFA, 2.5% TIS, 2.5% H_2O, 2.5% w/v DTT for 3 h (other peptides). The combined filtrates were collected into a round bottom flask and concentrated under reduced pressure. Peptides were always protected from light.

Ac-Asn-Arg-Val-Lys-Asp-Leu-Lys-Asn-Arg-Val-Lys-Asp-Leu-Lys-Lys-Glu-Leu-Ala-Arg-Leu-Glu-Lys-Glu-Asn-Glu-Ser-Leu-Lys-Arg-Glu-Ile-Ala-Tyr-Met-Glu-Lys-Glu-Leu-Ala-Arg-Leu-Glu-NH_2 (Ac-101A).

The synthesis was performed on 28 mg of Tentagel R RAM resin (0.18 mmol g^{-1}). After cleavage as described above, the crude peptide was precipitated by adding cold Et_2O (dropwise) and the resulting precipitate was decanted and dried (x3). Purification was conducted by semi-preparative HPLC with detection at 220 nm. Pure fractions were collected and lyophilized to afford pure peptide **Ac-101A** as a white solid (4.8 mg, 18%).

HPLC-MS: t_R: 4.82 min, 99% purity. **HRMS** (ESI+) (m/z) for $C_{224}H_{389}N_{69}O_{68}S$, $[M+H]^+$: 5169.000.

Ac-5-Asn-Arg-Val-Lys-Asp-Leu-Lys-Asn-Arg-Val-Lys-Asp-Leu-Lys-Lys-Glu-Leu-Ala-Arg-Leu-Glu-Lys-Glu-Asn-Glu-Ser-Leu-Lys-Arg-Glu-Ile-Ala-Tyr-Met-Glu-Lys-Glu-Leu-Ala-Arg-Leu-Glu-NH$_2$ (5-101A).

The synthesis was performed on 28 mg of Tentagel R RAM resin (0.18 mmol g^{-1}). After cleavage as described above, the crude peptide was precipitated by adding cold Et_2O (dropwise) and the resulting precipitate was decanted and dried (x2). Purification was conducted by analytical HPLC with detection at 340 and 486 nm. Pure fractions were collected and lyophilized to afford the pure peptide **5-101A** as a pale-yellow solid (3.1 mg, 11%).

HPLC-MS: t_R: 4.70 min, 98% purity. **HRMS** (ESI+) (m/z) for $C_{233}H_{396}N_{74}O_{71}SSe$, $[M+5H]^{5+}$: 1097.3790, $[M+6H]^{6+}$: 914.4846, $[M+7H]^{7+}$: 783.9881, $[M+8H]^{8+}$: 686.2394. **MALDI** (m/z): $[M+H]^{+}$: 5482.9403.

5(6)-carboxyfluorescein-Asn-Arg-Val-Lys-Asp-Leu-Lys-Asn-Arg-Val-Lys-Asp-Leu-Lys-Lys-Glu-Leu-Ala-Arg-Leu-Glu-Lys-Glu-Asn-Glu-Ser-Leu-Lys-Arg-Glu-Ile-Ala-Tyr-Met-Glu-Lys-Glu-Leu-Ala-Arg-Leu-Glu-NH$_2$ (fluorescein-101A).

The synthesis was performed on 28 mg of Tentagel R RAM resin (0.18 mmol g^{-1}). After cleavage as described above, the crude peptide was precipitated by adding cold Et_2O (dropwise) and the resulting precipitate was decanted and dried (x2). Purification was conducted by semi-preparative HPLC with detection at 220 and 450 nm. Pure fractions were collected and lyophilized to afford pure peptide **fluorescein-101A** as a pale-yellow solid (6.8 mg, 25%).

HPLC-MS: t_R: 4.85 min, 99% purity. **HRMS** (ESI+) (m/z) for $C_{243}H_{397}N_{69}O_{73}S$: $[M+5H]^{5+}$: 1097.9963, $[M+6H]^{6+}$: 915.1612, $[M+7H]^{7+}$: 784.5673, $[M+8H]^{8+}$: 686.6218. **MALDI** (m/z): $[M+H]^{+}$: 5485.8289.

Biotin-Arg-Glu-Ile-Ala-Tyr-Met-Glu-Lys-Glu-Leu-Ala-Arg-Leu-Glu-Asn-Arg-Val-Lys-Asp-Leu-Lys-Glu-Glu-Asn-Ala-Thr-Leu-Lys-Asp-Lys-Val-Lys-Thr-Leu-Lys-Asn-Arg-Val-Lys-Asp-Leu-Lys-NH_2 (biotin-101B).

The synthesis was performed on 28 mg of Tentagel R RAM resin (0.18 mmol g^{-1}). After cleavage as described above, the crude peptide was precipitated by adding cold Et_2O (dropwise) and the resulting precipitate was decanted and dried (x2). Purification was conducted by semi-preparative HPLC with detection at 220 and 280 nm. Pure fractions were collected and lyophilized to afford pure peptide **biotin-101B** as a pale-yellow solid (9.9 mg, 38%).

HPLC-MS: t_R: 4.53 min, 95% purity. **HRMS** (ESI+) (m/z) for $C_{230}H_{399}N_{69}O_{68}S_2$, $[M+5H]^{5+}$: 1057.5960, $[M+6H]^{6+}$:881.4974, $[M+7H]^{7+}$: 755.7128, $[M+8H]^{8+}$: 661.2490. **MALDI** (m/z): $[M+H]^{+}$:5283.8171.

SeNBD-Lys-Gln-Thr-Ser-Val-OH (15).

The synthesis was performed on 62.5 mg of 2-chlorotrityl polystyrene resin (0.4 mmol g^{-1}). After cleavage as described above, the crude peptide was precipitated by adding cold Et_2O (dropwise) and the resulting precipitate was decanted and dried (x2). Purification was conducted by semi-preparative HPLC with detection at 220 and 500 nm. Pure fractions were collected and lyophilized to afford pure peptide **15** as an intense orange solid (3.3 mg, 16%).

HPLC-MS: t_R: 4.52 min, 98% purity. **HRMS** (ESI+) (m/z) for $C_{35}H_{55}N_{11}O_{12}Se$, $[M+H]^{+}$: 902.3270, found, 902.3306. **MALDI** (m/z): $[M+H]^{+}$: 902.3270.

Ac-Ser-Ser-5-Glu-Ser-Asp-Val-OH (16).

The synthesis was performed on 28.2 mg of 2-chlorotirtyl polystyrene resin (0.6 mmol g^{-1}). After cleavage as described above, crude peptide was precipitated by adding cold Et_2O (dropwise) and the resulting precipitate was decanted and dried (x2). Purification was

conducted by preparative HPLC with detection at 220 and 480 nm. Pure fractions were collected and lyophilized to afford pure peptide **16** as an intense orange solid (0.9 mg, 5.5% yield).

HPLC-MS: t_R: 2.84 min, 98% purity. **HRMS** (ESI+) (m/z) for $C_{34}H_{47}N_{11}O_{18}Se$, $[M-H]^-$: 976.2193, found, 976.2098. **MALDI** (m/z): $[M+Na]^+$: 1000.2161.

Circular dichroism (CD). CD spectra were obtained at r.t. using a JASCO J-810 CD spectrometer. A range of 190-260 nm was scanned at a speed of 50 nm min^{-1}, with 1 nm data pitch, 1 nm bandwidth and 8 s response time. The presented spectra are averaged across 8 accumulations taken. Samples were prepared in phosphate buffered saline (PBS, pH 7.4). CD spectra were measured in 0.2 mm quartz cuvettes at the indicated concentrations. Raw data (mdeg) were converted to mean residue ellipticity (MRE; deg cm^2 $dmol^{-1}$ res^{-1}) by normalizing for path length, peptide concentration, and number of amide bonds.

Spectroscopy measurements. Optical properties were measured in a Synergy H1 BioTek spectrophotometer as previously described.[7] For liposome experiments, phosphatidylcholine (PC)-based liposomes were purchased from Liposoma, diluted in PBS and transferred into a black flat-bottom 96-well plate for optical measurements. All experiments were performed in triplicate, unless otherwise indicated.

Synaptosome preparation. The synaptosome extraction was performed according to published procedures with minor modifications.[8] The experiment was performed at 4°C, using the homogenization buffer (0.32 M sucrose, 1 mM HEPES at pH 7.4, complete EDTA-free protease inhibitor cocktail). Briefly, heterozygous *Psd95^{HaloTag}* mouse forebrain (approx 300 mg wet weight) was suspended in the homogenization buffer (3 mL) and the

homogenization was performed by 12 strokes with a teflon-glass homogenizer. A pellet was obtained by centrifugation (1,400 g, 10 min, 4°C) and resuspended in homogenization buffer (1.5 mL) with 3 strokes of the teflon-glass homogenizer. After centrifugation (700 g, 10 min, 4°C), the supernatants from both spins were pooled. Another centrifugation (14,000 g, 10 min, 4°C) was carried out and the pellet containing synaptosomes was collected. The pellet was resuspended in homogenization buffer (0.6 mL) and underwent a final sucrose gradient centrifugation (0.85M/1.0M/1.2M sucrose, 2 mL each, 83,000 g, 2 h, 4°C). The purified synaptosomes were collected from the band between 1 M and 1.2 M sucrose.

Fluorescence microscopy. Single-molecule and super-resolution imaging was performed on a Total Internal Reflection Fluorescence microscope (TIRFM) described previously.[7] Briefly, collimated laser light at 515 nm (Cobolt Fandango-300 DPSS Laser System, Cobalt, Sweden), 561 nm (Cobolt DPL561-100 DPSS Laser System, Cobalt, Sweden), and 638 nm (Cobolt MLD Series 638-140 Diode Laser System, Cobolt AB, Solna, Sweden) was aligned and directed parallel to the optical axis at the edge of a 1.49 NA TIRF Objective (CFI Apochromat TIRF 60XC Oil), mounted on an inverted Nikon TI2 microscope. The microscope was fitted with a perfect focus system to auto-correct the z-stage drift during imaging. Fluorescence collected by the same objective was separated from the returning TIR beam by a dichroic mirror DI02-R514 (Semrock, Rochester, NY, USA) for 515 nm and Di01-R405/488/561/635 (Semrock, Rochester, NY, USA) for 561 nm and 638 nm, and was passed through appropriate filters (515 nm: BLP01-561R, FF01-607/36, 561 nm: LP02-568-RS, FF01-587/35, 638 nm: FF01-432/515/595/730-25, LP02-647RU-25). Fluorescence was then passed through a 2.5× beam expander and recorded on an EMCCD camera (Delta Evolve 512, Photometrics) operating in frame transfer mode (EMGain = 11.5 e^-/ADU and 250 ADU/photon). Each pixel was 103 nm in length. Images were recorded with an exposure time of 50 ms with ~500 W cm^{-2} illumination. The microscope was automated using the

open-source microscopy platform Micromanager. Borosilicate glass coverslips (20 × 20 mm, VWR International) were cleaned using an Ar plasma cleaner (Zepto, Diener) for 30 min to remove any fluorescent residues. Frame-Seal slide chambers (9 × 9 mm^2, Biorad) were affixed to the glass to create a well in which samples were added. 101A-101B peptides. The plasma-cleaned coverslips were incubated with BSA-biotin (1 mg mL^{-1}, 50 µL) for 10 min. Slides were washed three times with PBS and incubated with AF647-labelled streptavidin (16.6 nM) for 10 min. Following three PBS washes, the anchor peptide **biotin-101B** (100 nM) was incubated for 10 min. Finally, after three PBS washes, the imaging peptide **5-101A** (100 nM) was applied to the surface. Samples were imaged using 515 nm excitation (for **5-101A**) and 638 nm (for AF647) under wash-free conditions. Fluorescence images were acquired for 50 frames with 50 ms exposure on both channels. Liposomes. 50 µL of poly-L-lysine (70,000-150,000 Da, Sigma-Aldrich) was added to the coverslip on the inside of the chamber and incubated for at least 30 minutes, before being washed with PBS three times. Liposomes (Liposoma) were diluted 100-fold to a concentration of 0.04 mg mL^{-1} and incubated on the coverslip for 10 min before being washed three times with PBS. BODIPY-FL or compound **13** were added at either 10 nM or 1 µM and imaged with 515 nm excitation for 50 frames. To determine the photobleaching lifetimes, the liposomes imaged with 1 µM compound **13** µM or BODIPY-FL were segmented and their intensity profiles over the frame stack were fit to an exponential decay. Only fits with an $R^2 > 0.70$ were considered (Python code available at: https://doi.org/10.5281/zenodo.7275931). Synaptosomes. The synaptosome preparation was incubated with SiR HaloTag ligand (40 µM) for 10 min on ice. The resulting solution was diluted 10 times in PBS and incubated on a plasma-cleaned coverslip for 3 min. Slides were then washed three times with PBS, and either imaging peptide **15** or **16** (500 nM) was added. SiR was excited using 638 nm laser light and 500 frames were collected, and peptide **15** with 561 nm and peptide **16** with 515 nm irradiation for 5000 frames and 10,000 frames respectively. Peptide-PAINT images were analyzed

using Fiji (Java 8 2017 release) and single localizations were processed using the Peak Fit function of the Fiji GDSC SMLM plugin, using a signal strength threshold of 20, and a precision threshold of 50 nm. Using an approach previously published,[9] clusters of PSD-95 localization events indicative of whole synaptosomes were defined using GDBSCAN,[10] compiled in Python 3.8 (sklearn v0.24.2) using epsilon = 1 pixels and a minimum points threshold of 100 to remove random localizations. For each localization within their respective clusters, the distance to the nearest neighboring localization (defined as the nearest neighbor distance) was first determined, and from this, the mean nearest neighbor distance (ND_{ave}) for each cluster was calculated. The number of neighbors (NN) for each localization was then determined by counting localizations within 5 x ND_{ave} for each cluster. Nearest neighborhood images were generated by plotting each localization as a circle, the color of which was weighted on the NN coefficient. The resolution of each synaptosome was calculated as a function of the localization fitting precision and the average nearest neighbor distance in accordance with previously published methods[11] (Python code available at: https://doi.org/10.5281/zenodo.7275947). The overall resolution of the images was calculated using Fourier Ring Correlation analysis (Python code available at: https://doi.org/10.5281/zenodo.7275952).

Statistical analysis. Statistical differences were analyzed two-sided unpaired *t* tests using Graphpad software. Independent experiments were performed at least three times, unless otherwise mentioned.

NMR spectra

Compound **5** (DMSO-d_6)

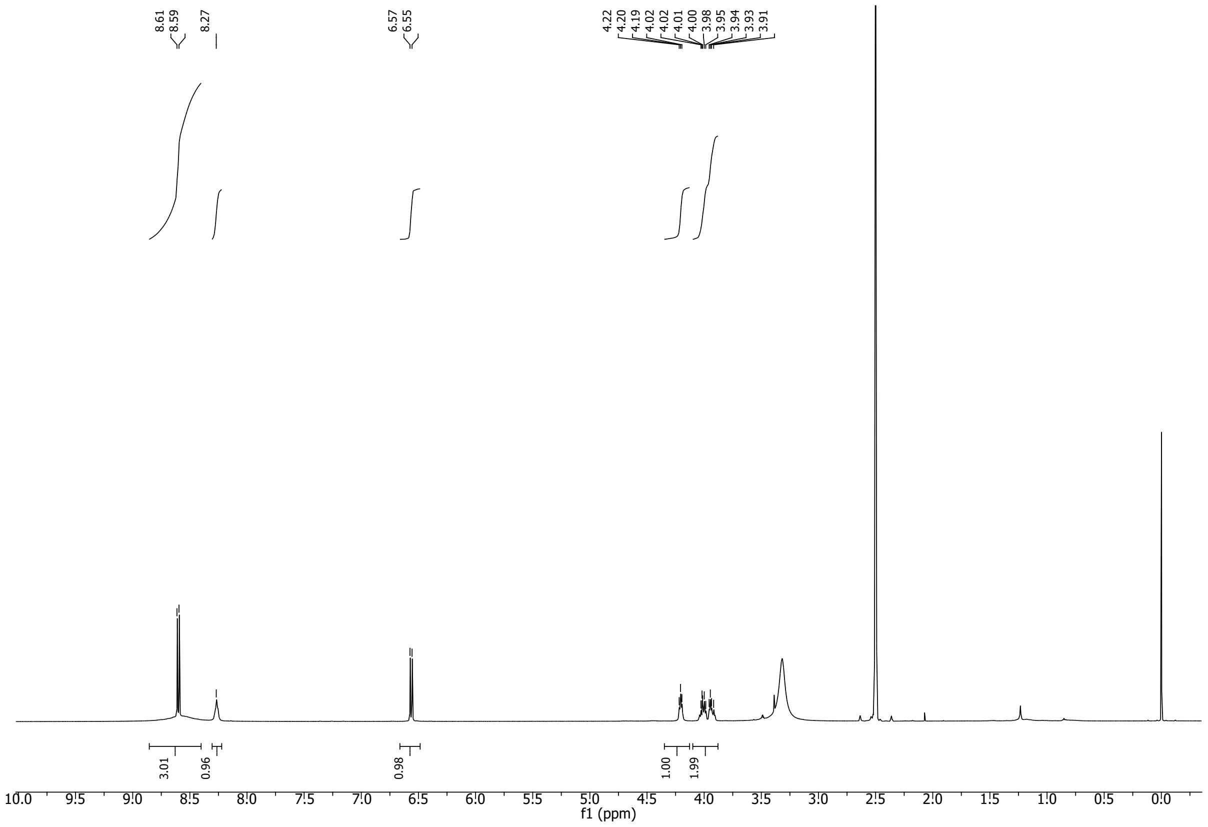

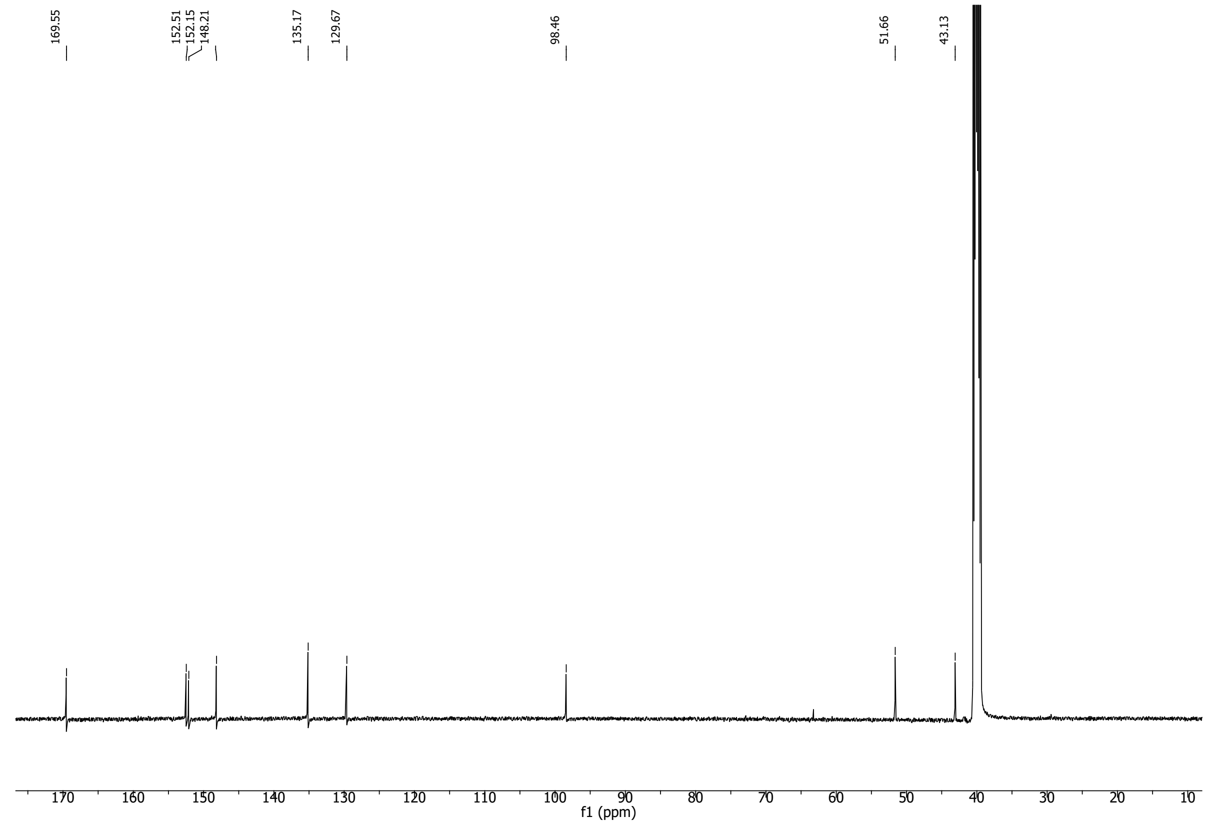

Compound **6** (MeOD)

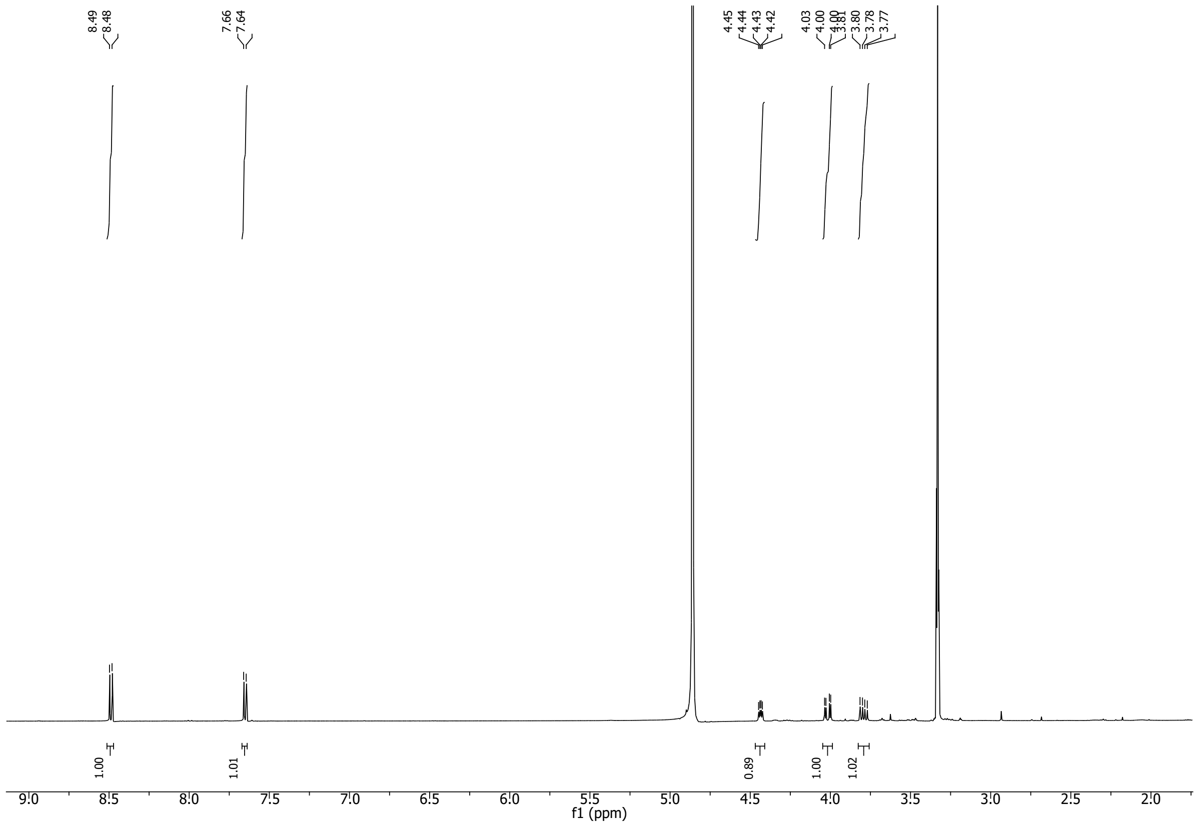

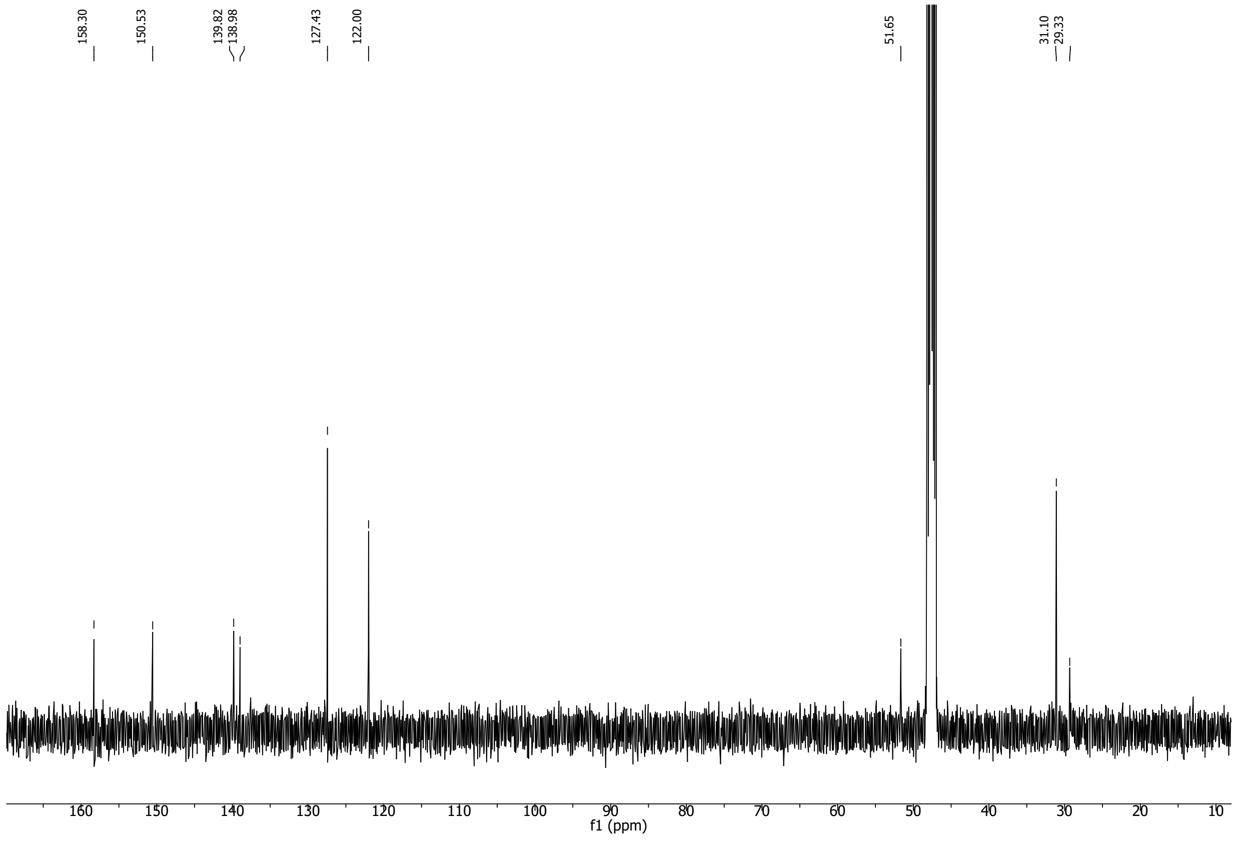

Compound **7** (DMSO-d_6)

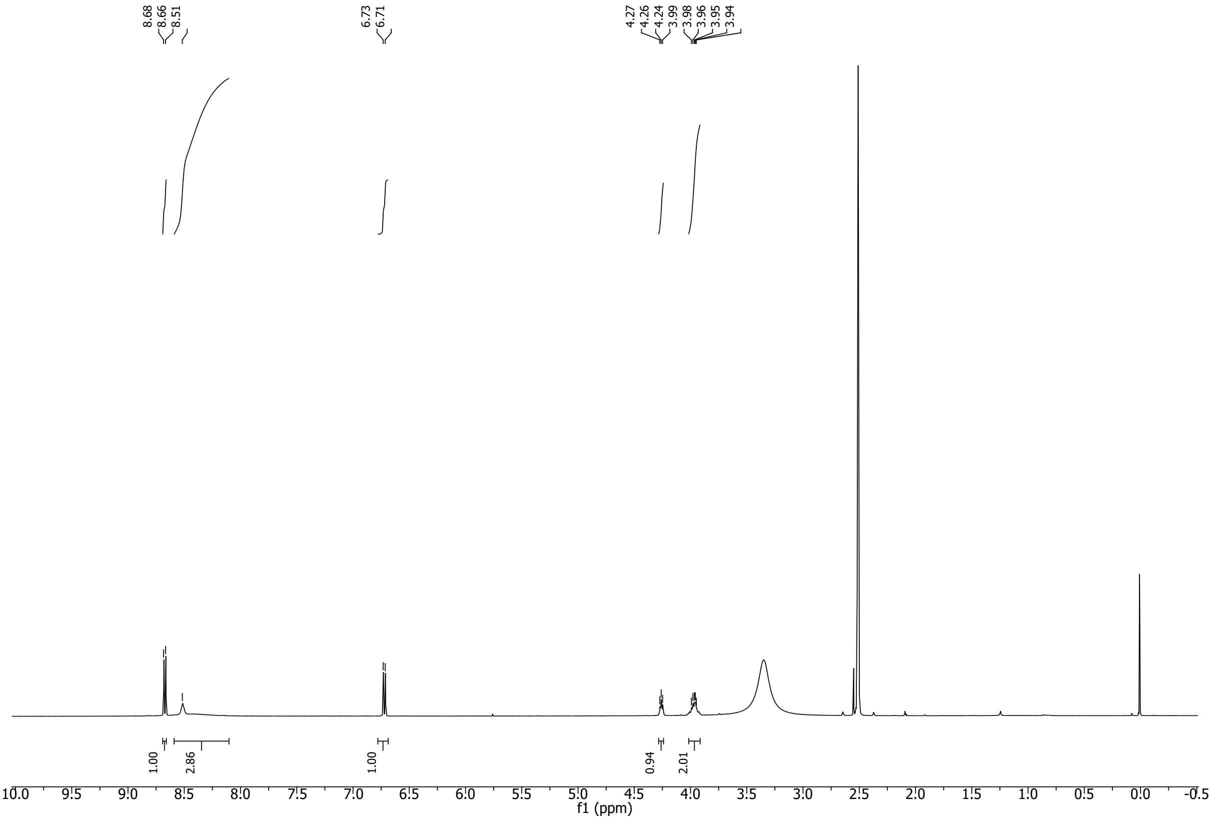

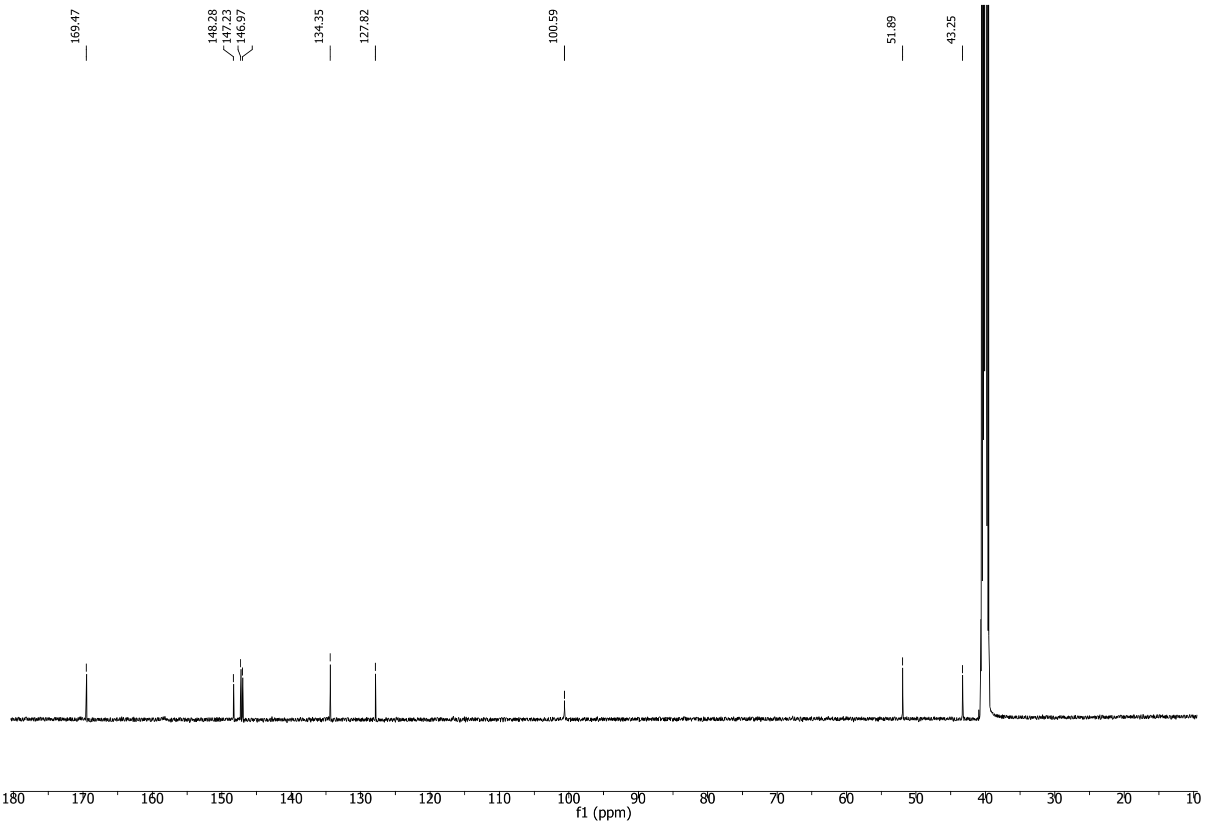

Compound **8** (MeOD)

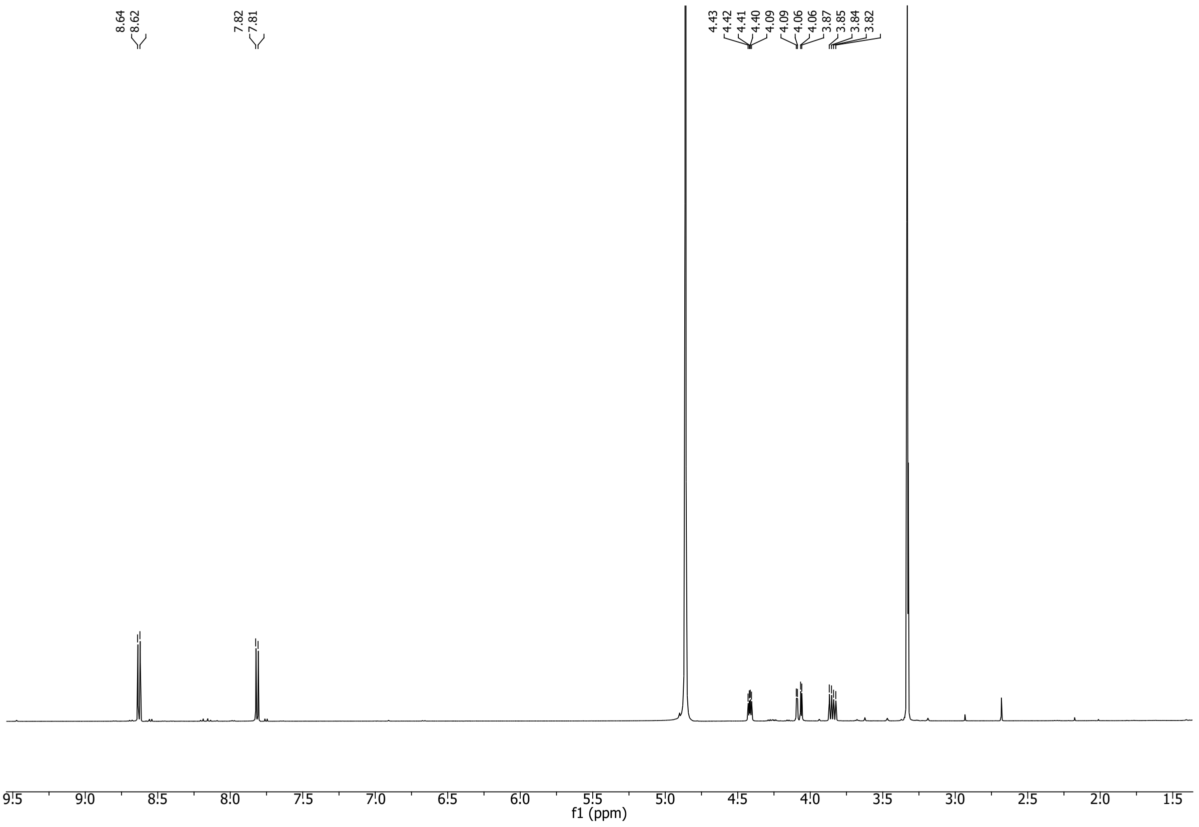

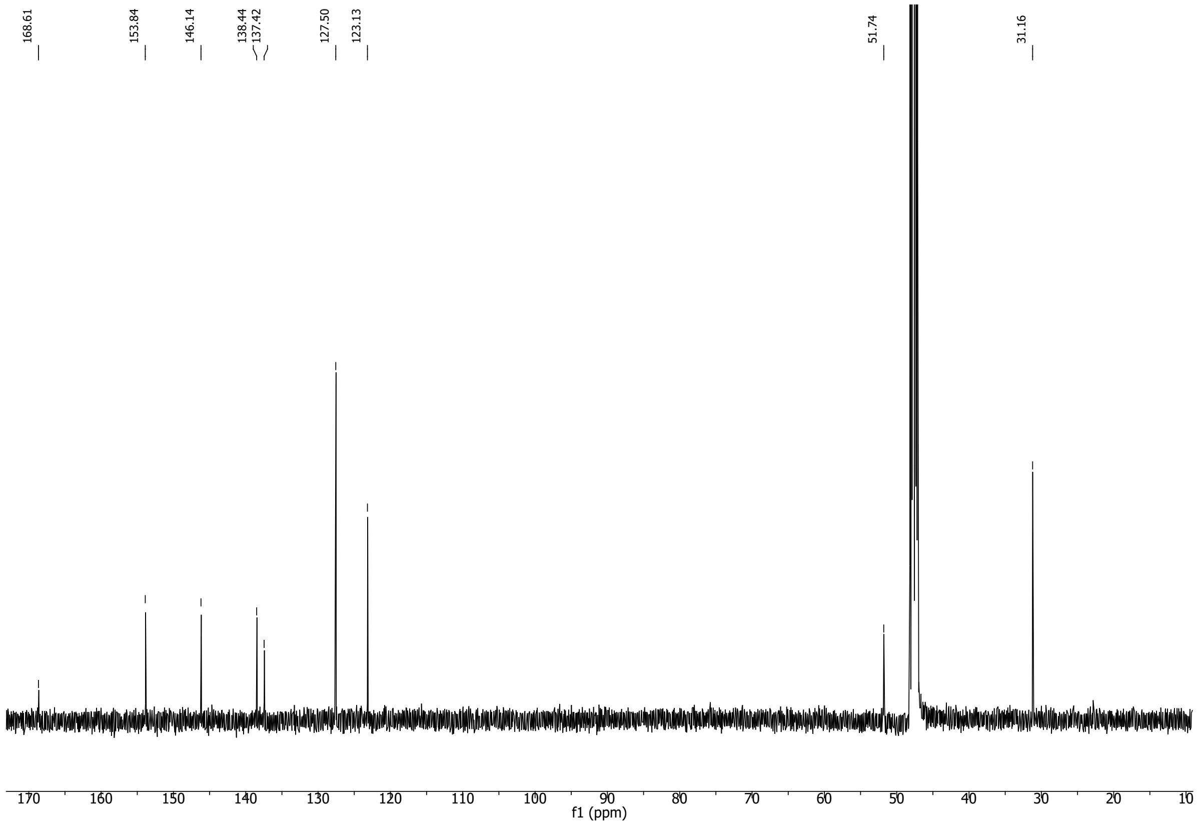

Compound **9** (DMSO-d_6)

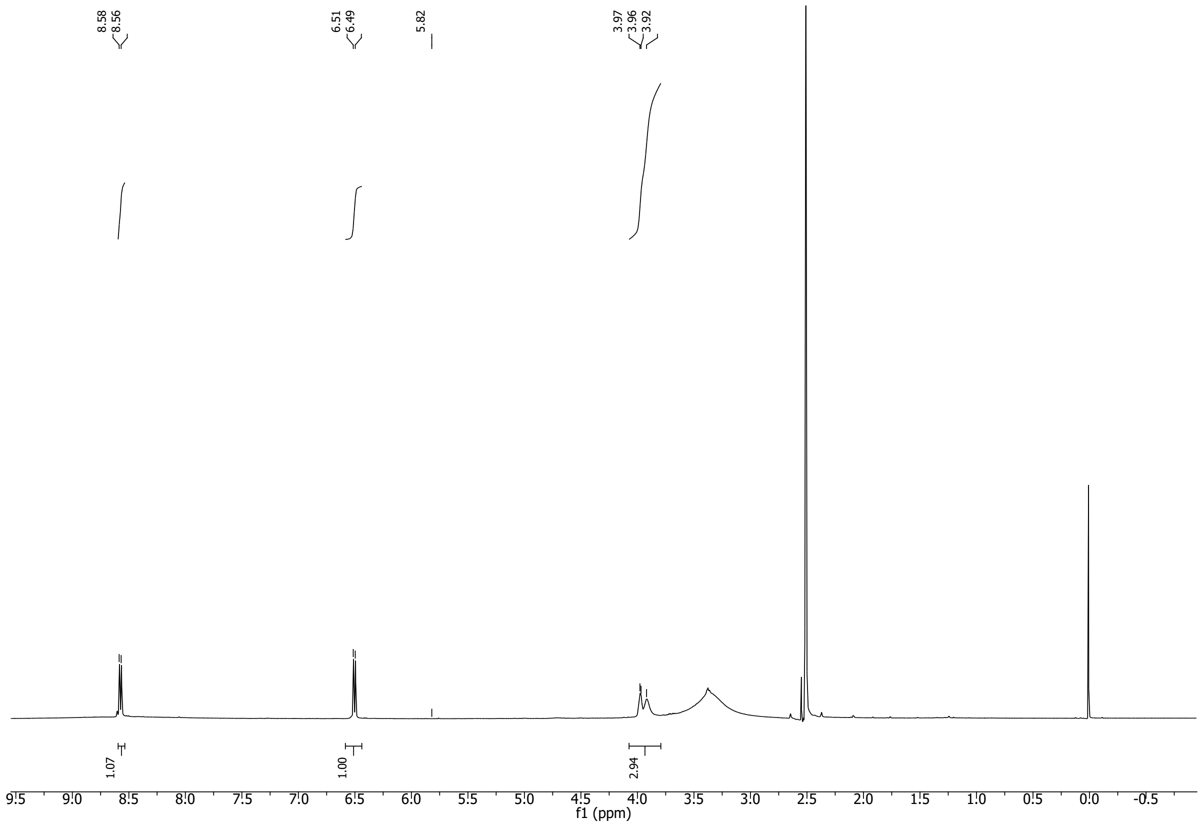

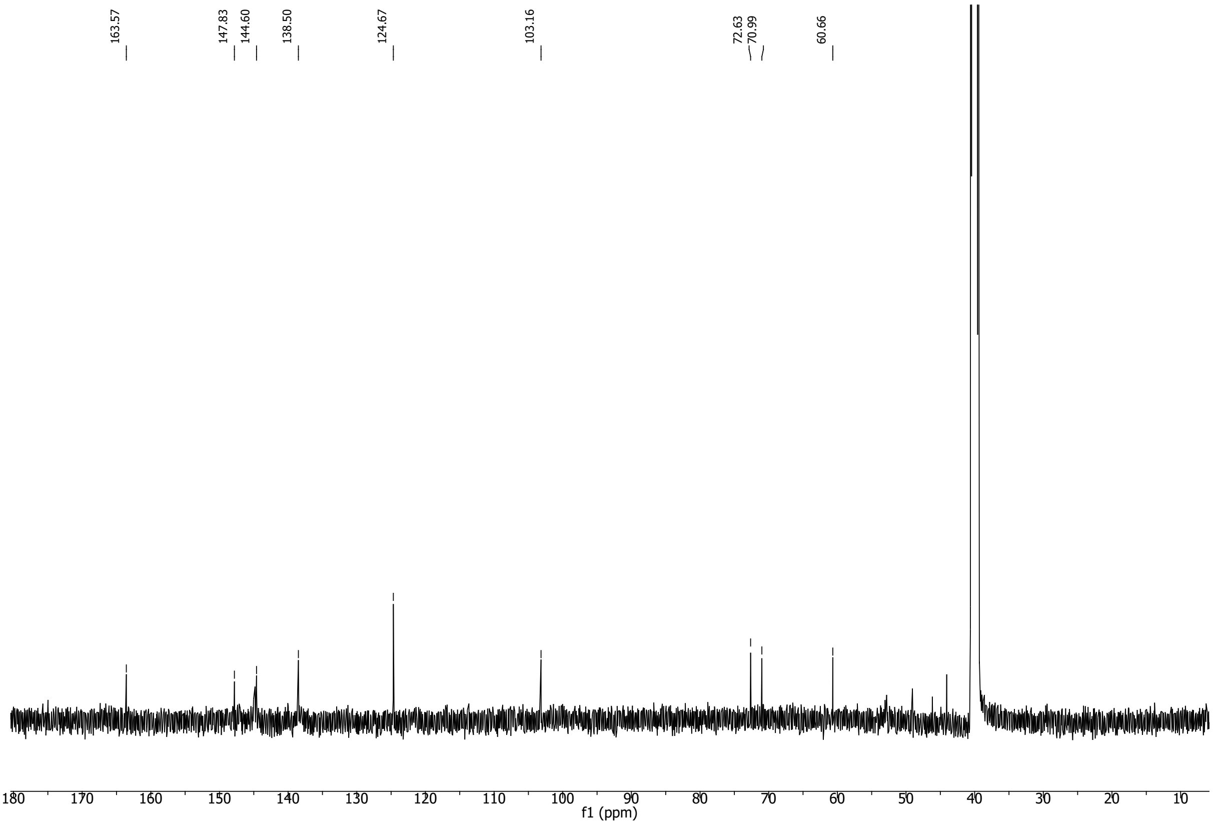

Compound **10** D_2O (contains 1,4-dioxane traces)

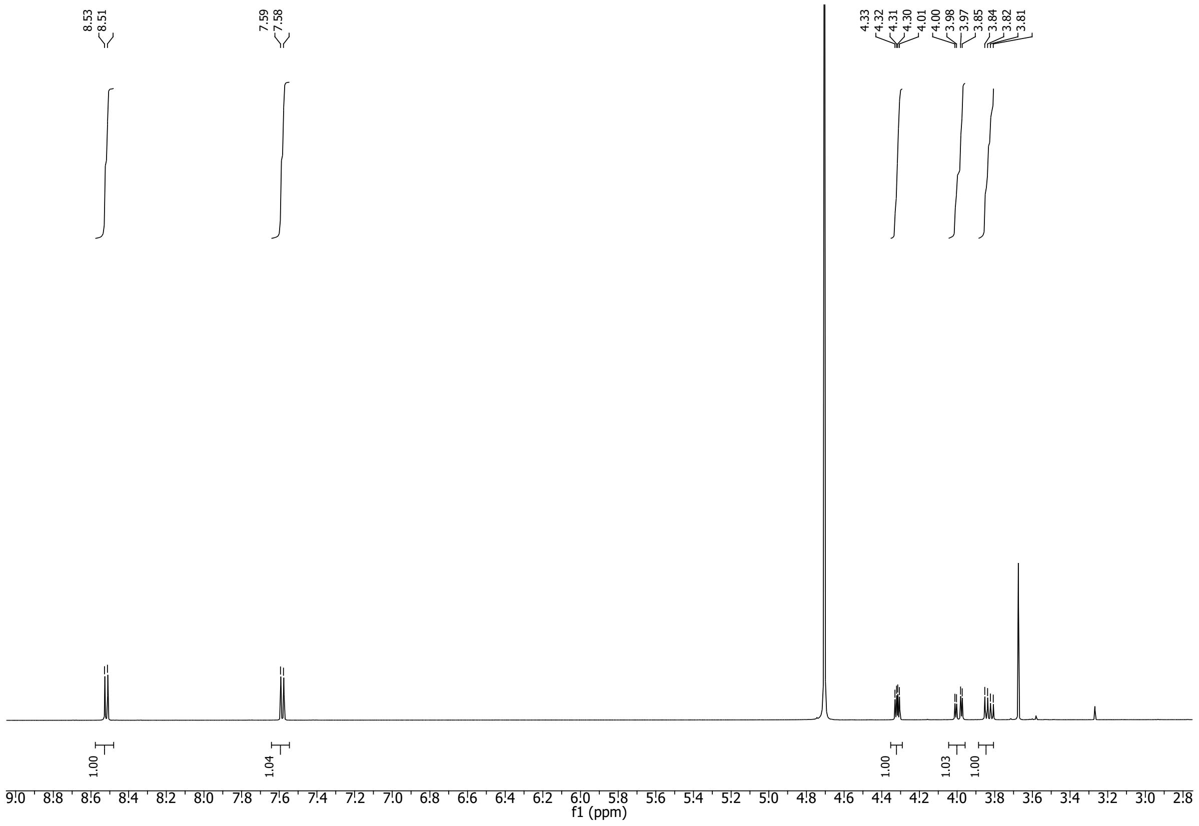

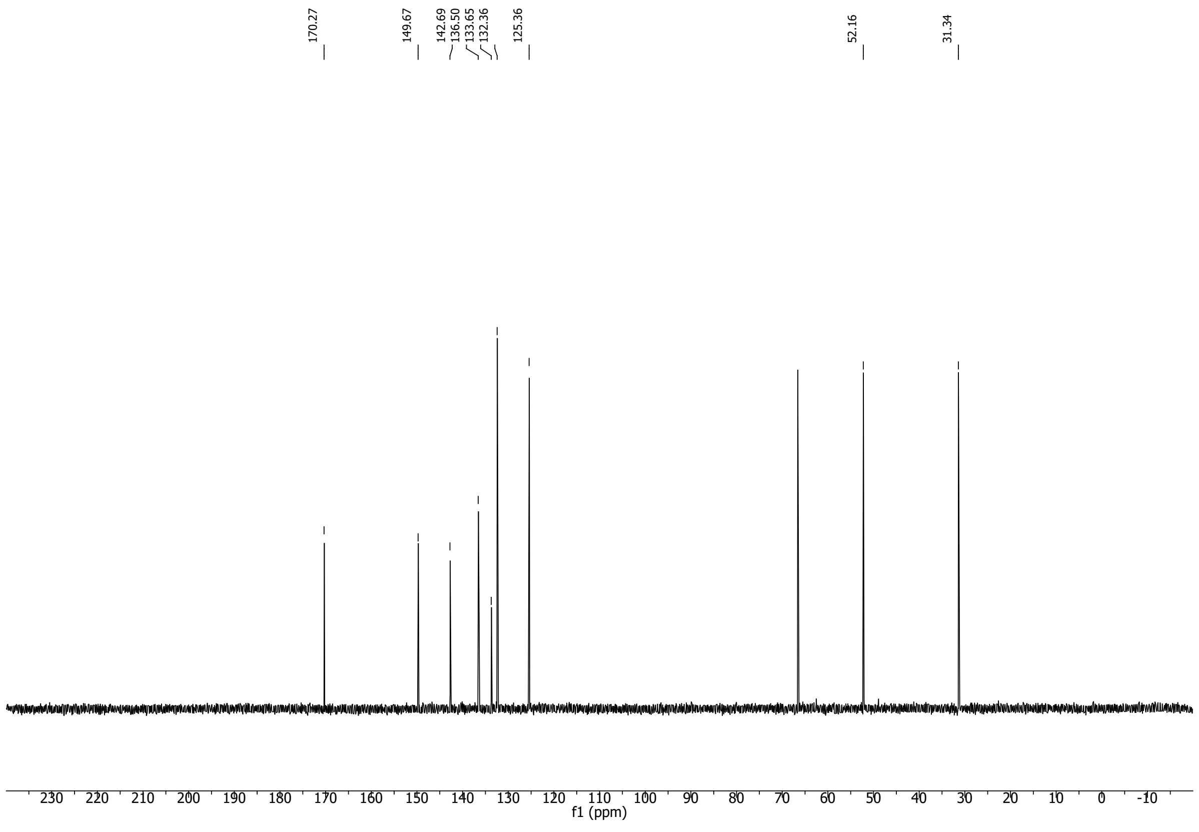

Compound **11**(MeOD)

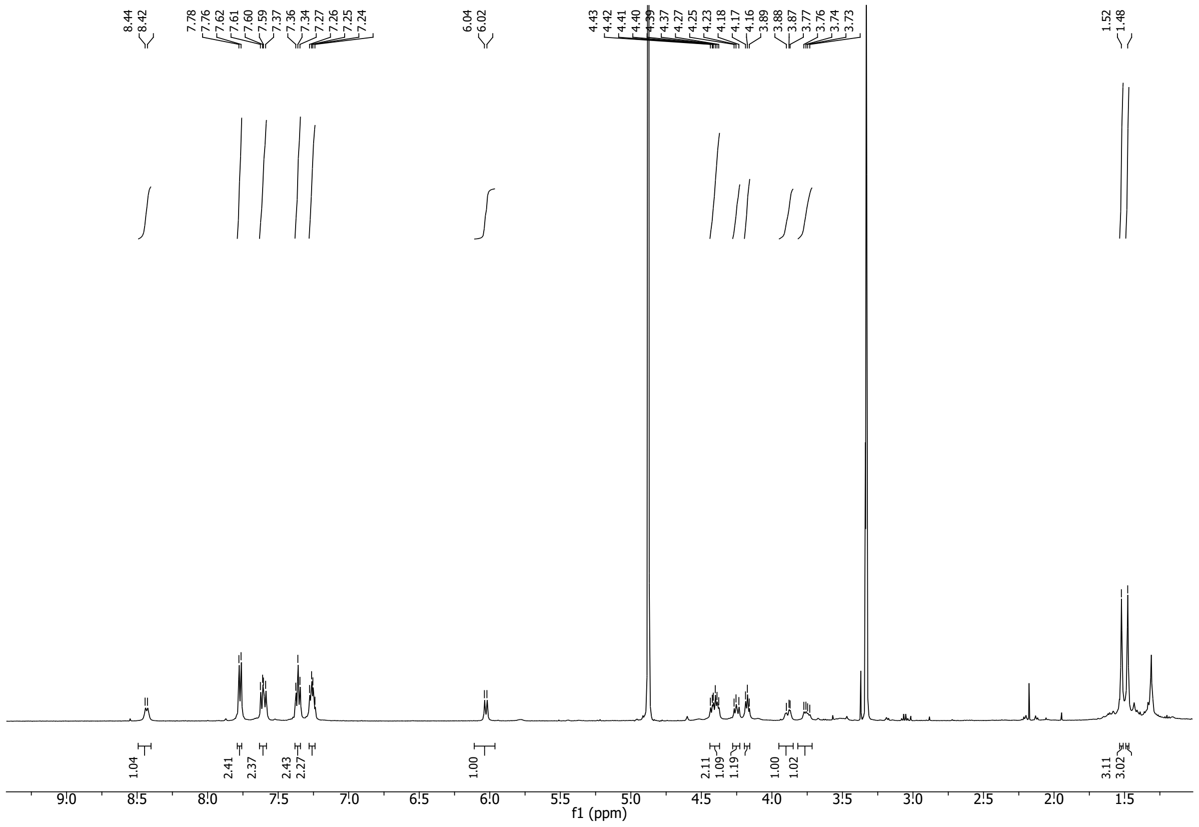

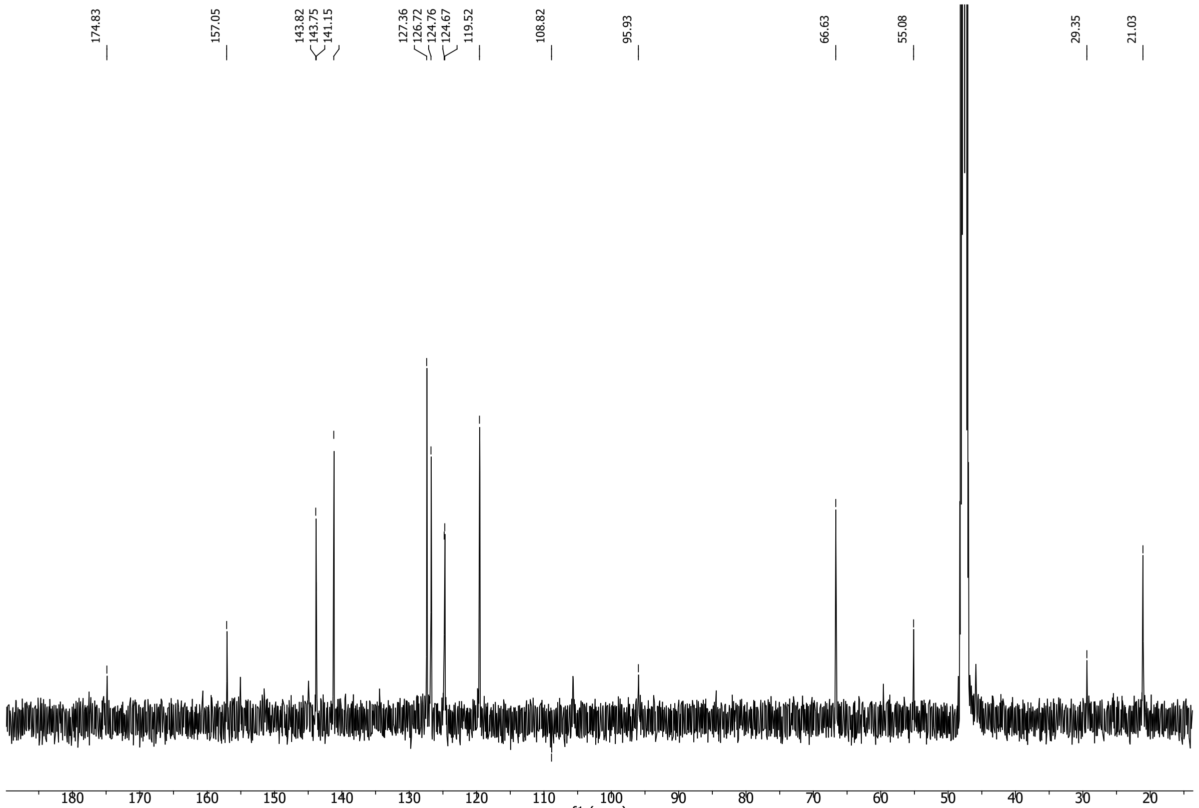

Compound **12** (DMSO-d_6)

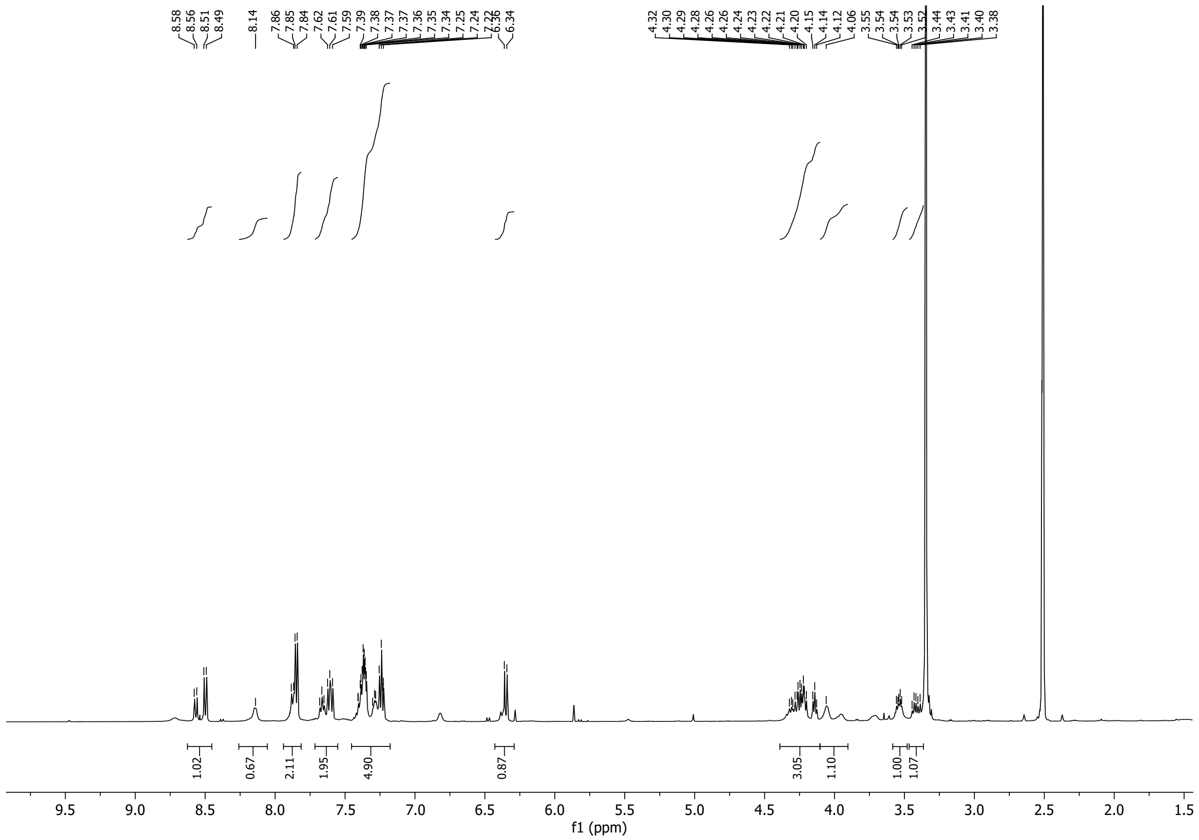

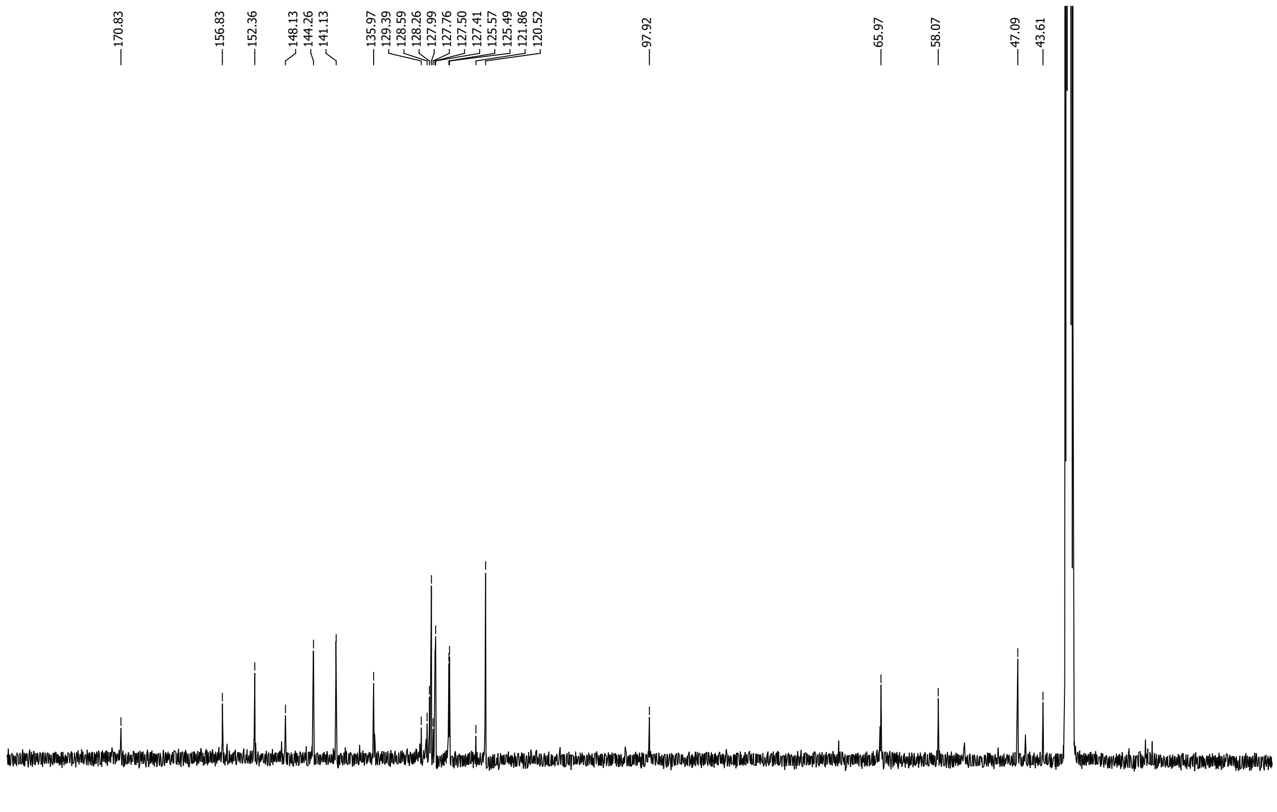

Compound **13** ($CDCl_3$)

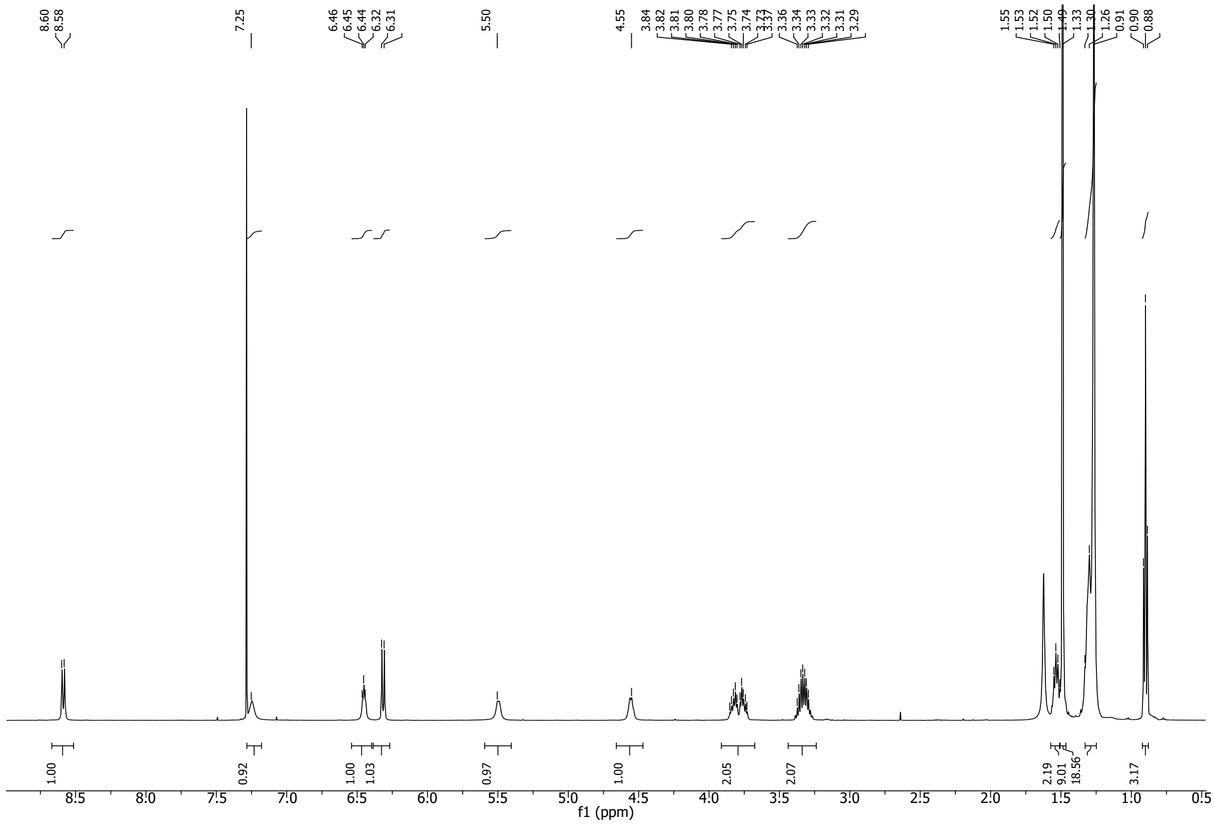

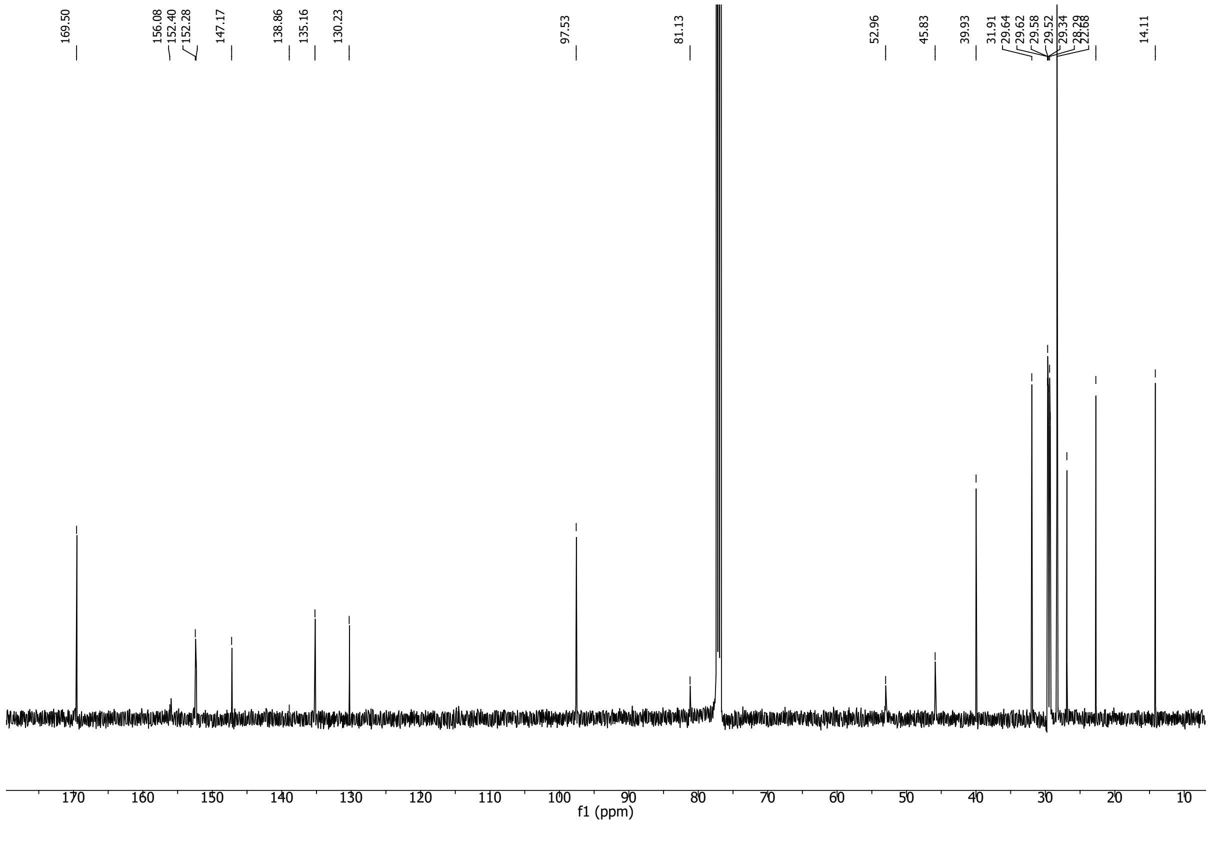

Compound **14** ($CDCl_3$)

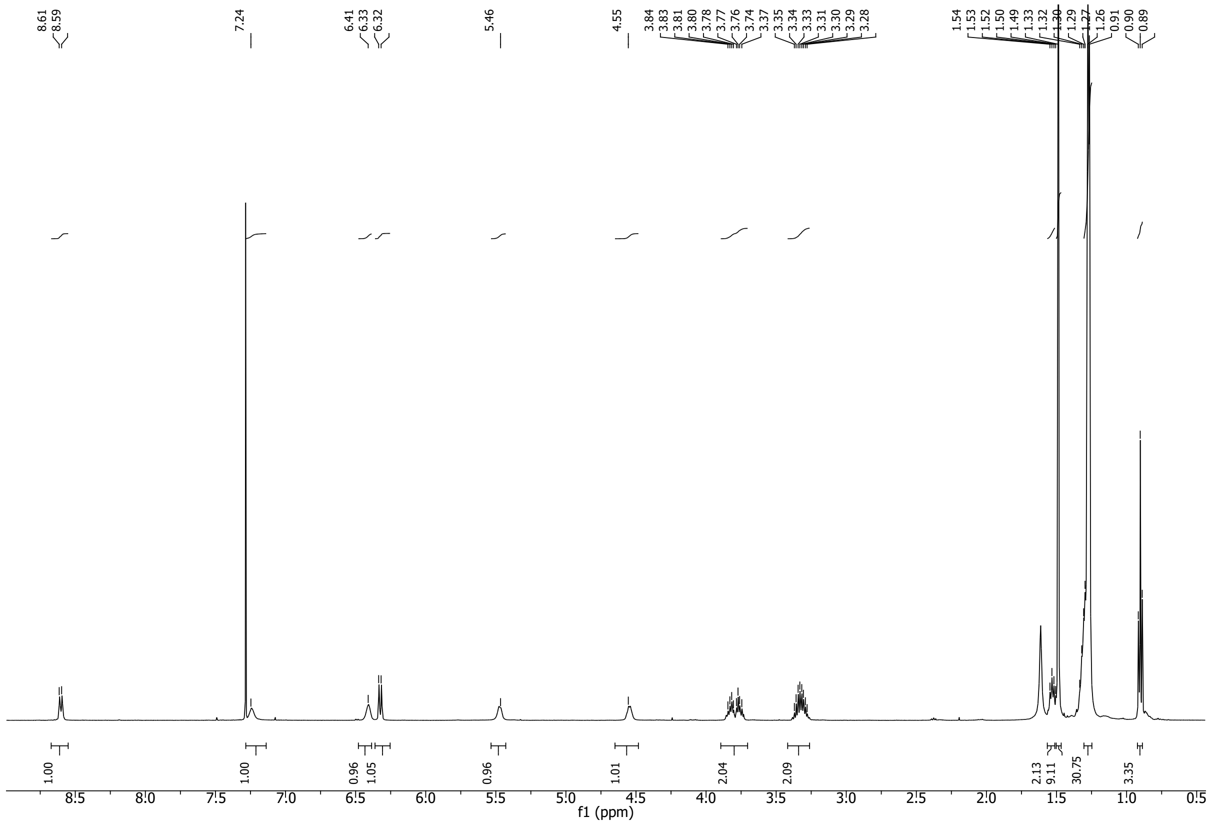

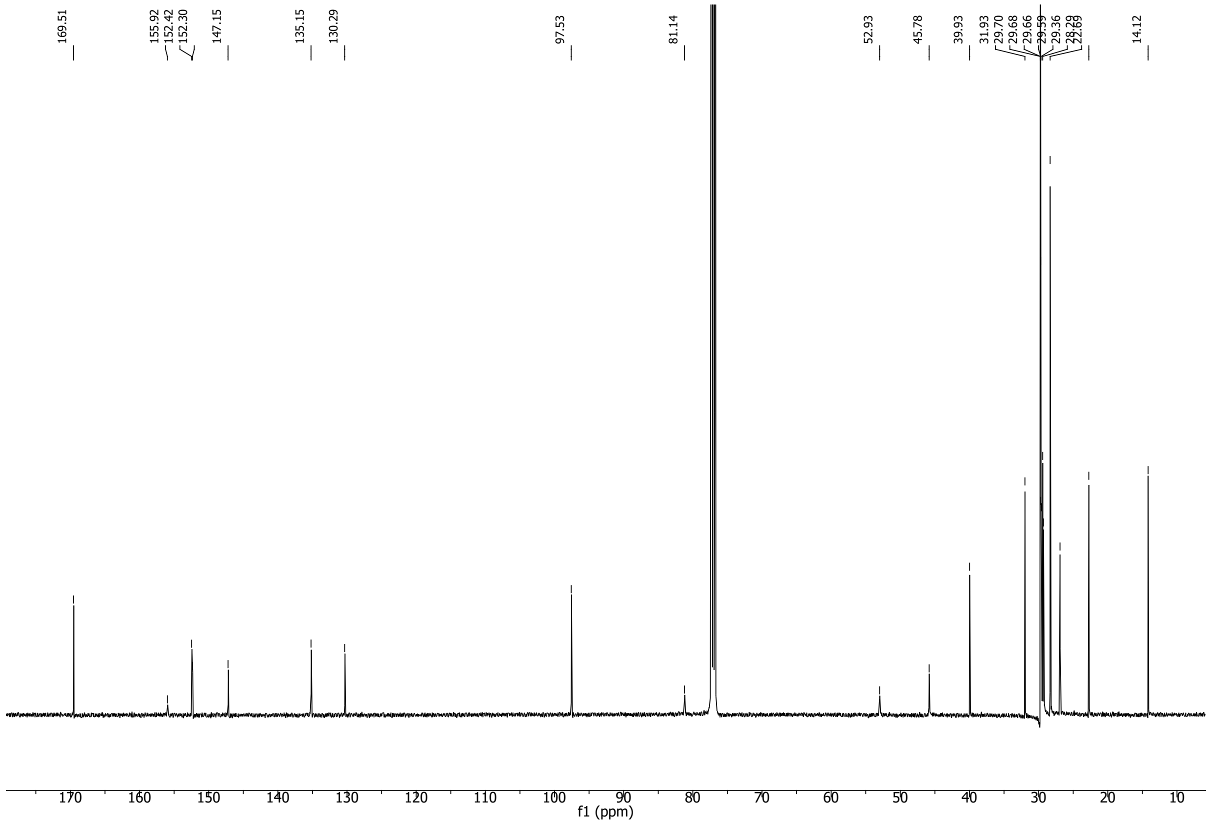

MALDI spectra

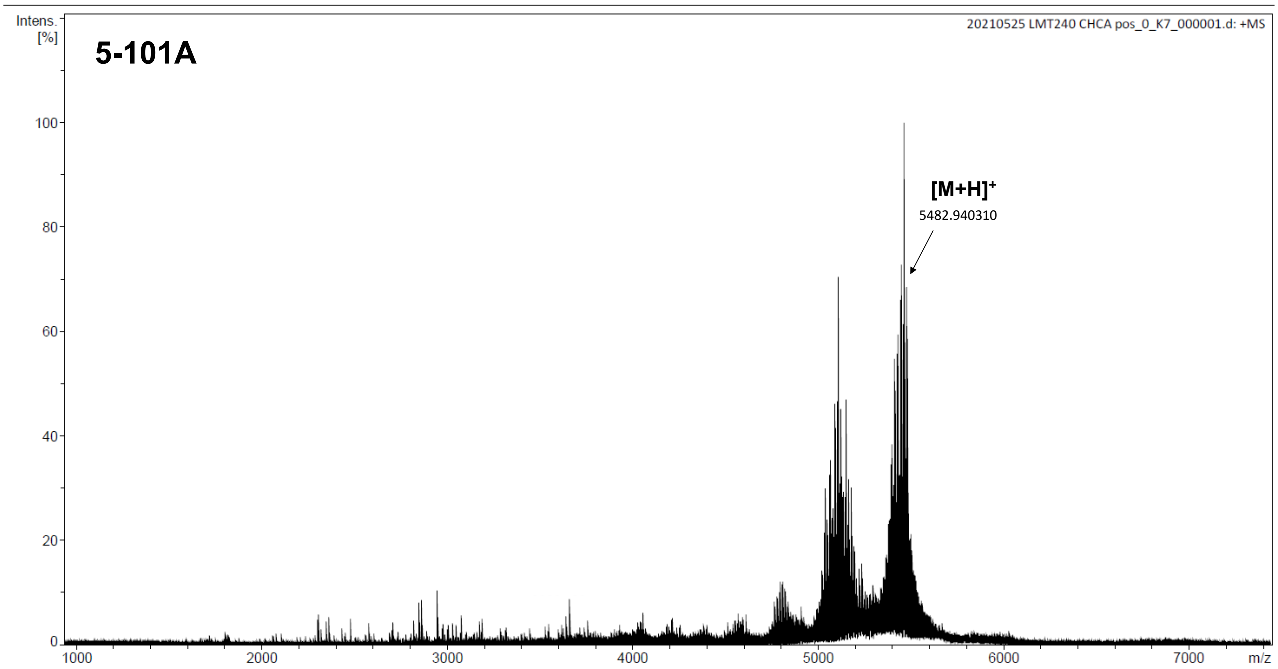

MALDI (m/z): $[M+H]^+$: 5482.9403.

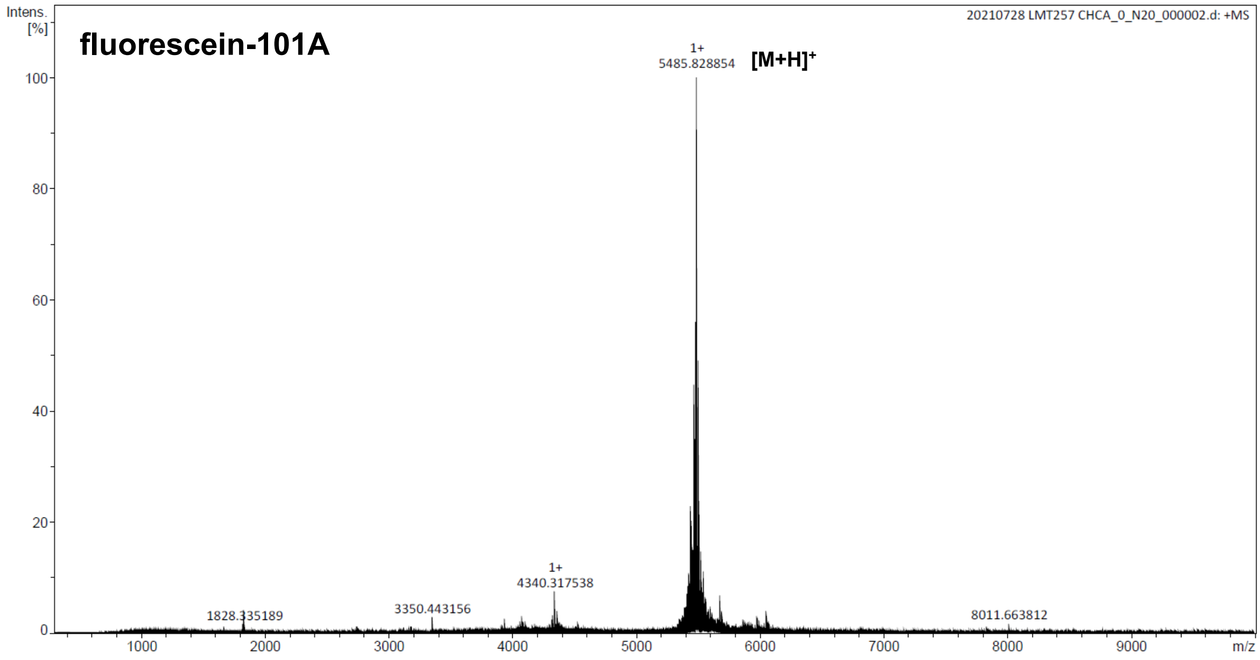

MALDI (m/z): $[M+H]^+$: 5485.8289.

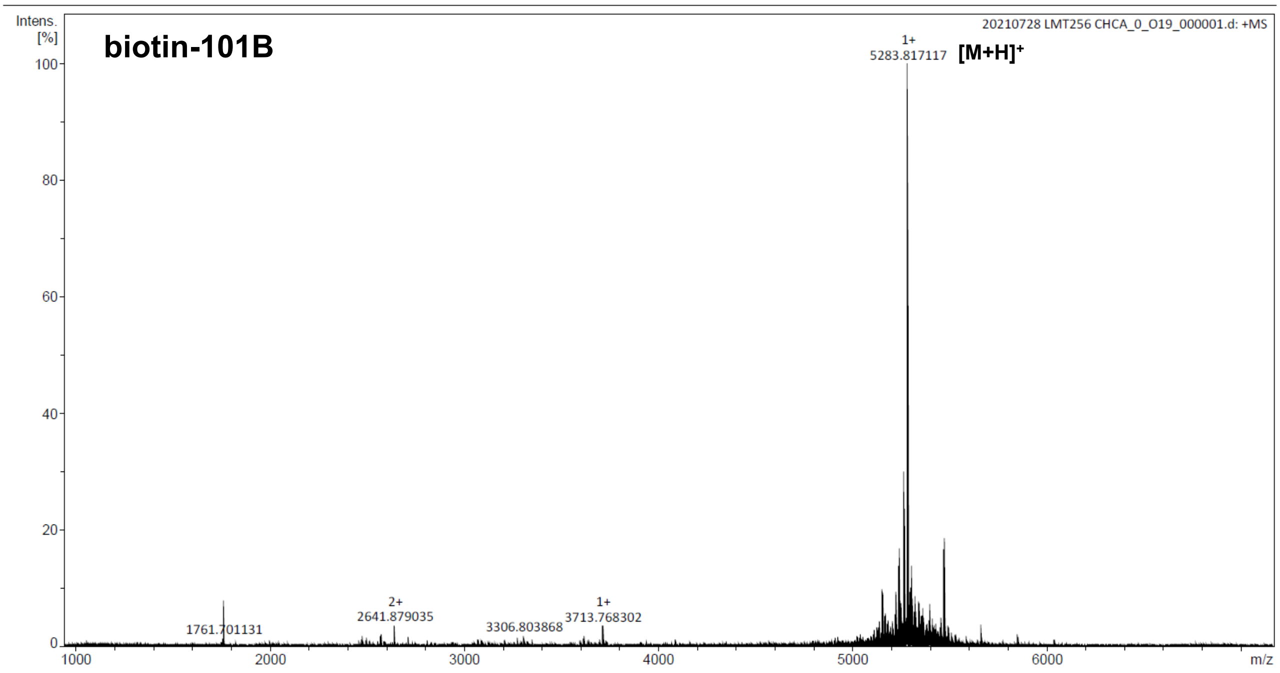

MALDI (m/z): [M+H]$^+$:5283.8171.

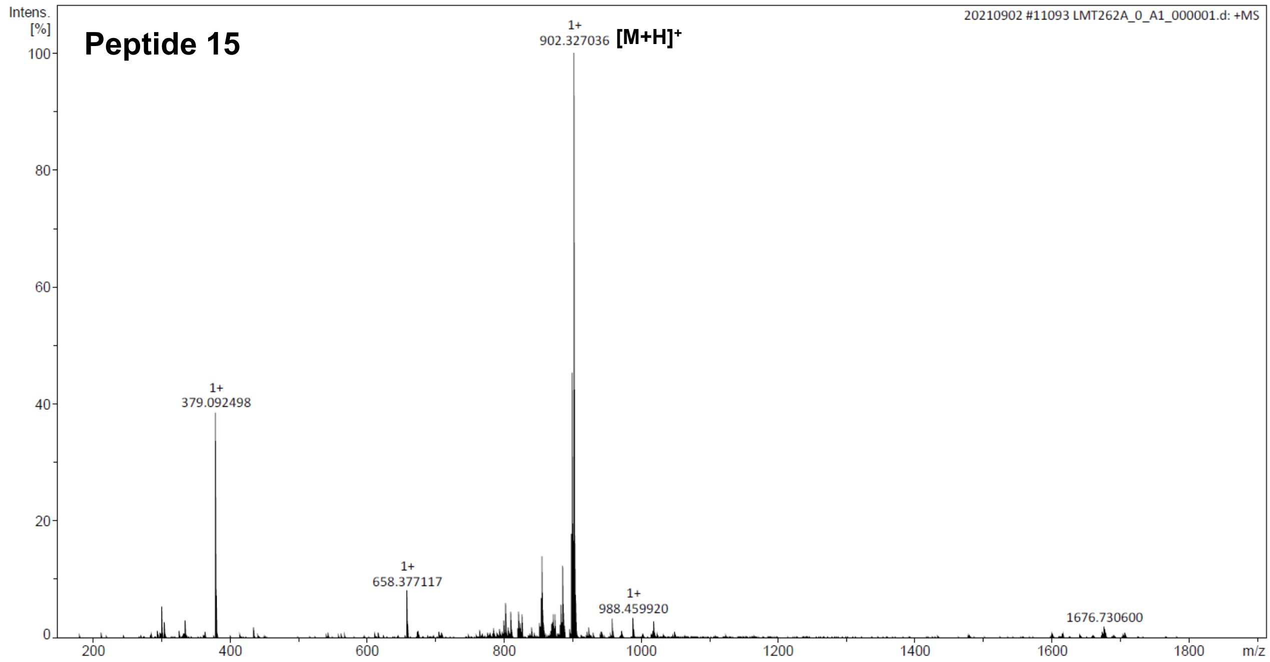

MALDI (m/z): [M+H]$^+$: 902.3270.

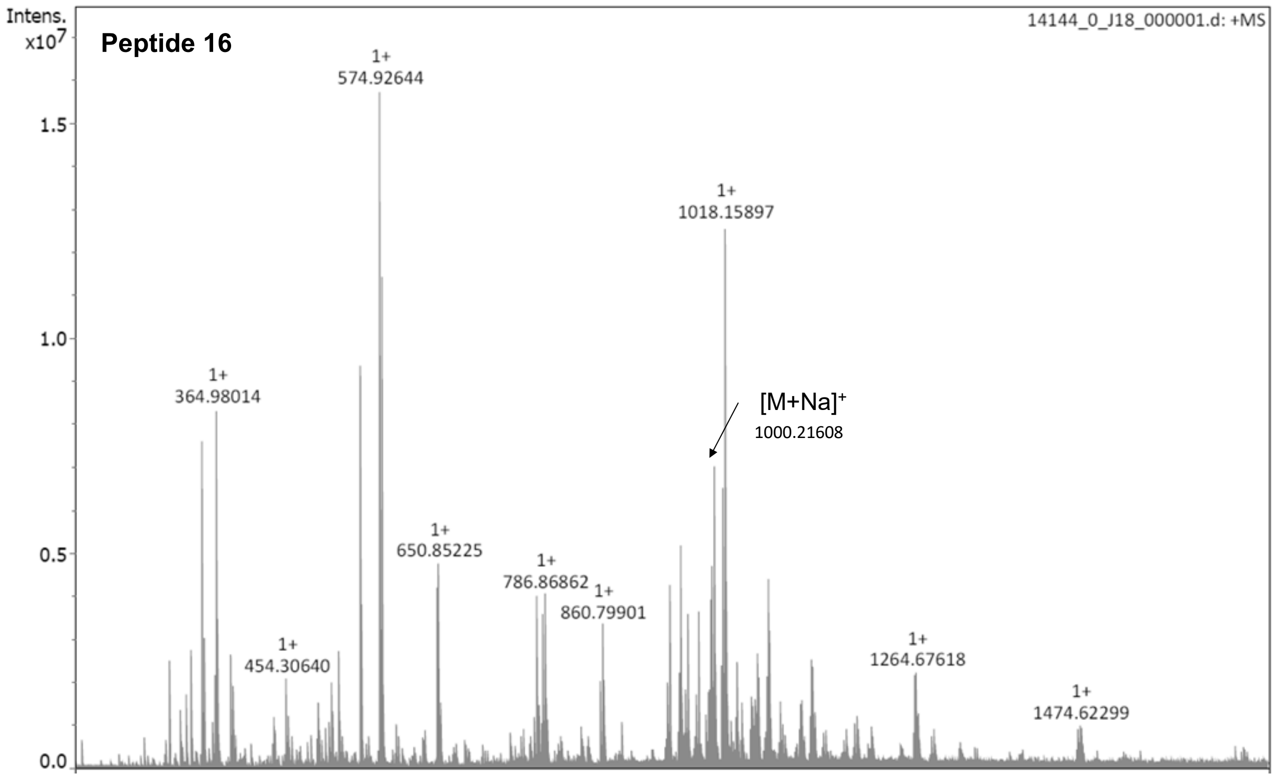

MALDI (m/z): $[M+Na]^+$: 1000.2161.

Supplementary References

[1] M. Cooper, A. Ebner, M. Briggs, M. Burrows, M. Gardner, R. Richardson, R. J. West, *J Fluoresc* **2004**, *14*, 145–150.

[2] B. Rudat, E. Birtalana, S. B. L. Vollrath, D. Fritz, D. K. Kölmel, M. Nieger, U. Schepers, K. Müllen, H.J. Eisler, U. Lemmer, S. Bräse, *Eur J Med Chem* **2011**, *46*, 4457–4465.

[3] K. Kolmakov, V.N. Belov, J. Bierwagen, C. Ringemann, V. Müller, C. Eggeling, S.W. Hell, *Chem Eur J* **2010**, *16*, 158–166.

[4] J. B. Grimm, B. P. English, H. Choi, A. K. Muthusamy, B. P. Mehl, P. Dong, A. T. Brown, J. Lippincott-Schwartz, Z. Liu, T. Lionnet, L. D. Lavis, *Nat Methods* **2016**, *13*, 985–988.

[5] S. Abou-Hatab, V. A. Spata, S. Matsika, *J Phys Chem A* **2017**,*121*, 1213–1222.

[6] T. Förster, G. Hoffmann, *Z Phys Chem* **1971**, *75*, 63–76.

[7] S. Benson, A. Fernandez, N. D. Barth, F. de Moliner, M. H. Horrocks, C. S. Herrington, J. L. Abad, A. Delgado, L. Kelly, Z. Chang, Y. Feng, M. Nishiura, Y. Hori, K. Kikuchi, M. Vendrell, *Angew Chem Int Ed* **2019**, *58*, 6911-6915; *Angew Chem* **2019**, *131*, 6985–6989.

[8] R. K. Carlin, D. J. Grab, R. S. Cohen, P. Siekevitz, *J Cell Biol* **1980**, *86*, 831–845.

[9] H. D. MacGillavry, Y. Song, S. Raghavachari, T. A. Blanpied, *Neuron* **2013**, *78*, 615–622.

[10] J. Sander, M. Ester, H. P. Kriegel, X. Xu, *Data Min Knowl Discov* **1998**, *2*, 169–194.

[11] T. Gould, V. Verkhusha, S. Hess, *Nat Protoc* **2009**, *4*, 291–308.
